# Fractional neural sampling as a theory of spatiotemporal probabilistic computations in neural circuits

**DOI:** 10.1038/s41467-022-32279-z

**Published:** 2022-08-05

**Authors:** Yang Qi, Pulin Gong

**Affiliations:** 1 School of Physics, University of Sydney, Sydney, NSW 2006 Australia; 2 Institute of Science and Technology for Brain-Inspired Intelligence, Fudan University, Shanghai, 200433 China; 3 Key Laboratory of Computational Neuroscience and Brain-Inspired Intelligence (Fudan University), Ministry of Education, Shanghai, China; 4 MOE Frontiers Center for Brain Science, Fudan University, Shanghai, 200433 China

**Keywords:** Network models, Dynamical systems

## Abstract

A range of perceptual and cognitive processes have been characterized from the perspective of probabilistic representations and inference. To understand the neural circuit mechanism underlying these probabilistic computations, we develop a theory based on complex spatiotemporal dynamics of neural population activity. We first implement and explore this theory in a biophysically realistic, spiking neural circuit. Population activity patterns emerging from the circuit capture realistic variability or fluctuations of neural dynamics both in time and in space. These activity patterns implement a type of probabilistic computations that we name fractional neural sampling (FNS). We further develop a mathematical model to reveal the algorithmic nature of FNS and its computational advantages for representing multimodal distributions, a major challenge faced by existing theories. We demonstrate that FNS provides a unified account of a diversity of experimental observations of neural spatiotemporal dynamics and perceptual processes such as visual perception inference, and that FNS makes experimentally testable predictions.

## Abstract (web summary)

Dynamics of neural circuits mapping brain functions such as sensory processing and decision making, can be characterized by probabilistic representations and inference. The authors elaborate the role of spatiotemporal neural dynamics for more efficient performance of probabilistic computations.

## Introduction

Humans and other animals operate in a world that is noisy and ambiguous. Moreover, it has been widely observed that the firing activity of neural systems is inherently stochastic and neural responses to stimuli exhibit large variability within and across trials^1–3^. Such exogenous and endogenous randomness naturally leads to the view that neural computations are carried out in a probabilistic way^4–7^. Indeed, a host of brain functions from sensory processing^8,9^ and cognitive tasks^10,11^ to motor behaviors^12^ have been successfully characterized from the perspective of probabilistic inference. The success of probabilistic accounts of these brain functions raises the fundamental question of how neural activity represents probability distributions and how neural circuits implement probabilistic inference based on that representation.

Two prominent types of models have been proposed to understand the neural basis of probabilistic representations and computations. One type is based on neural population activity that encodes the parameters of probability distributions (e.g., probabilistic population codes) such as the variance of Gaussian distributions^4,13^. The other type of model employs sampling-based probabilistic representations^14–19^. Probabilistic representations in these models are mainly based on the variability or fluctuations of neural activity with Poisson or Gaussian statistics. However, empirical evidence has increasingly demonstrated that neural fluctuations occur at multiple scales with heavy-tailed, non-Gaussian statistics^20,21^, and that such fluctuations unfold in both time and space to give rise to rich, complex spatiotemporal dynamics of neural circuits^22,23^.

**Fig. 1:**
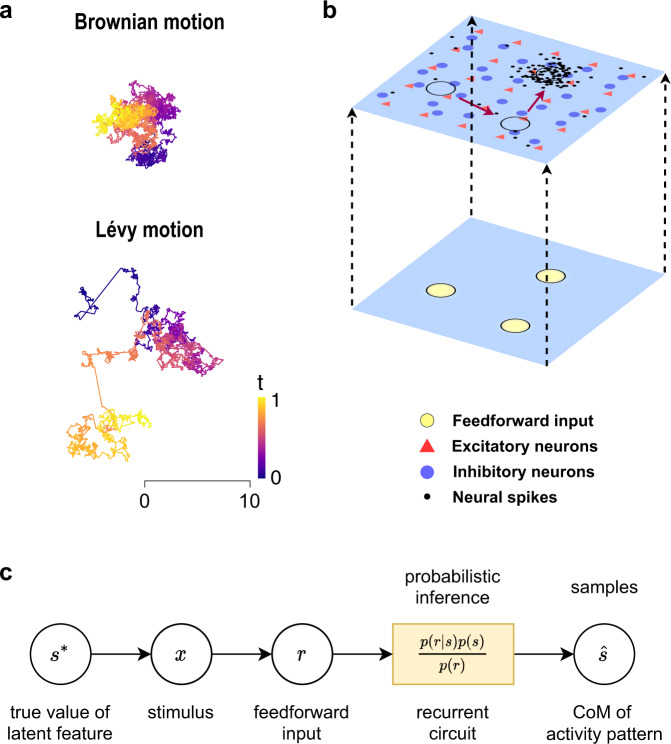
Complex spatiotemporal dynamics of neural circuits implement probabilistic computations. **a** Typical sample path of a Brownian motion and that of a Lévy motion. The latter consists of clusters of short step sizes that are intermittently interspersed by long jumps. In contrast, the Brownian motion lacks such long jumps. Color indicates time. **b** Schematic diagram of the two-dimensional E-I spiking neural circuit. The circuit consists of recurrently connected excitatory and inhibitory neurons spanning a two-dimensional feature space (top layer), receiving multiple feedforward inputs (bottom layer). The spiking activity pattern (dots) emerging from the circuit exhibits rich, complex spatiotemporal dynamics. As indicated by the red arrow, the localized activity pattern intermittently switches between different parts of the neural circuit (black circles) and can implement sampling-based probabilistic computations. **c** Schematic diagram of a generative process for sampling-based probabilistic inference in our model.

Here we argue that these rich spatiotemporal dynamics enable a new type of sampling-based probabilistic neural computation. This probabilistic computation offers a unique perspective on the functional role of spatiotemporal neural dynamics and provides a solution to a long-standing challenge of reliably sampling complex probability distributions such as multimodal distributions. Sampling multimodal distributions is fundamentally important for processing natural environments replete with multiple, distributed salient patches (i.e., modes)^24,25^, and for learning and inference^26^. This form of processing remains a major challenge in previous studies, because Gaussian fluctuations or noise used in existing models essentially implement Brownian-motion-based Markov chain Monte Carlo (MCMC) sampling and its variants^14,15,17^. Due to the lack of ‘large jumps’ in Brownian motion (Fig. 1a), when faced with multimodal distributions with far-apart modes, neural samplers are unable to traverse through low-probability regions; such samplers are thus prone to be trapped in one local mode and lack the capacity of freely switching from one mode to another^27^.

We illustrate our probabilistic computation theory through a biophysically realistic, spatially-extended spiking neural circuit (Fig. 1b), demonstrating that population activity patterns (i.e., neural ensembles) emerging from the circuit possess large intrinsic fluctuations at multiple spatial and temporal scales as empirically observed. Rather than Brownian motion, the activity patterns propagating across the neural circuit with such large fluctuations exhibit clusters of short step-sizes that are intermittently interspersed by long jumps (Fig. 1a), with the movement step sizes showing heavy-tailed, non-Gaussian (Lévy) statistics^28^. These intermittent, long jumps inherent in Lévy motion enable the activity patterns to adaptively and freely switch between different modes of multimodal distributions, thus sampling these distributions with great efficiency. Besides these heavy-tailed Lévy motions in space, our sampling approach exploits certain temporal oscillatory components that play a fundamental role in speeding up the sampling process. We demonstrate that our sampling-based representation accounts for key response properties of neural circuits such as the reduction of neural variability after stimulus onset^3,29^ and theta oscillations (3–8 Hz) accompanied by 1/f activity as widely observed during environmental sampling tasks^30^, and that the sampling dynamics of the neural circuit are the underlying mechanism for perceptual switching^31^.

We further elucidate that the key spatial and temporal properties of the sampling-based representation implemented in the circuit model can be characterized by a stochastic differential equation with a fractional order derivative, which generalizes the notion of differentiation to fractional orders and captures heavy-tailed Lévy motions occurring at multiple scales^28^. We thus develop a mathematical model of our sampling approach; due to the fractional nature revealed by this mathematical model, we term our sampling approach fractional neural sampling (FNS). Based on this mathematical model, we illustrate the essential algorithmic properties of FNS for efficiently sampling multimodal distributions and further validate predictions from the mathematical model in our spiking neural circuit, thus revealing how and why FNS works at both the circuit implementation and algorithmic levels.

FNS-based probabilistic inference produces estimates about the mean and variance of probability distributions that match those of optimal Bayesian inference. However, due to the presence of small movements occasionally interrupted by large ones in FNS, it naturally generates switching-like behaviors and the resultant multi-modes of the estimate probability distributions. Our FNS-based inference thus provides a mechanistic account of why there exists bimodality in estimate distributions, as observed in a recent psychophysics study of visual perception inference^32^; this experimental result is not explained by conventional models of probabilistic inference. Our FNS-based inference model further makes quantitative predictions about how statistics of perceptual estimate distributions are related to stimulus contrast; these predictions are consistent with reanalysis of the existing experimental data. Dynamical switching between different activity patterns representing either multiple external sensory inputs^33^ or multiple choices in decision making^34,35^ have been widely observed, suggesting that FNS-based probabilistic computations could be of general applicability to understanding brain functions ranging from sensory processing to decision making.

## Results

We first illustrate how FNS works based on a biophysically realistic circuit model of spiking neurons and then reveal the circuit mechanism underlying the emergence of key spatial and temporal properties of FNS. Second, we formulate FNS by using a mathematical model derived from fractional diffusion formalisms, based on which we elucidate the computational properties of FNS for sampling multimodal distributions. We then validate these properties in the spiking neural circuit model. Finally, we illustrate how FNS-based probability inference can be implemented in the spiking neural circuit with the prior embedded in recurrent synaptic weights, and demonstrate that our model provides a novel account of visual perceptual inference as observed in experimental studies.

### Circuit implementation of fractional neural sampling

**Fig. 2:**
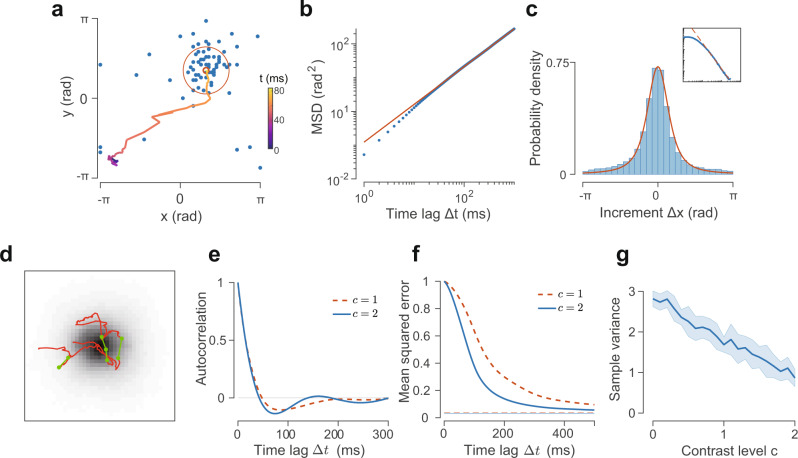
Spatial and temporal properties of FNS. **a** Snapshot of the localized activity pattern when the I–E ratio *ξ* = *ξ*_*c*_ = 3.4. Dots denote spikes during a 5 ms period. The small circle marks the center-of-mass (CoM) of the pattern and the large circle represents one standard deviation of the spike locations. The line shows the CoM trajectory of the pattern over the previous 80 ms, with color indicating time. **b** Mean squared displacement (MSD) of the trajectory of the activity pattern as a function of time lag. The red line indicates a power-law fit, MSD(Δ*t*) ∝ Δ*t*^*η*^, with the diffusion exponent *η* = 1.18. **c** Distribution of trajectory increments. The red line indicates a fitted symmetric *α*-stable distribution, with the tail index *α* = 1.28. Inset: distribution of the trajectory increment on a log–log scale. **d** A typical sample trajectory overlaid on top of the sampled probability density. Large jumps with lengths greater than 0.25 are highlighted with connected green dots. **e** Autocorrelation of the sample trajectories for different stimulus contrast levels. Averaged across 100 trails of 10 s duration. **f** Mean squared error of the sample mean decreases with time lag, with a small baseline due to quenched noise (randomness in synaptic connectivity). **g** Dependence of the sample variance on input contrast; shades indicate the standard deviation of the sample variance across 20 trials.

We consider a biophysically realistic spiking neural circuit model of excitatory and inhibitory neurons that incorporates experimentally established properties of the cortex, such as distance-dependent synaptic connectivity^36,37^ and correlated excitatory and inhibitory synaptic inputs^38^ (Fig. 1b, see “Methods”). The neural circuit spans a two-dimensional feature space^39^ with its *x* and *y* coordinates representing feature values such as orientation (angle) and color (hue), respectively, both ranging from −*π* to *π*. The circuit model exhibits a rich repertoire of dynamical activity states including asynchronous activity (i.e., disordered state) and localized propagating waves (i.e., ordered state)^40^, depending on the relative strengths of synaptic inhibition and excitation (characterized by an I–E ratio *ξ*; see “Methods”). Near the phase transition (*ξ* = *ξ*_*c*_ = 3.4) between these two states, localized activity patterns with rich, complex spatiotemporal dynamics emerge and behave like random walkers, exhibiting clusters of small movement step-sizes that are intermittently interspersed by long jumps (Fig. 2a). Such intermittent motion of the localized patterns can be characterized by Lévy motion (Fig. 1a), a type of non-equilibrium motion that has been shown to be essential for animals to optimally search for spatially distributed food^41,42^, for T-cells to efficiently find target pathogens in brain explants^43^, and for optimally transporting energy in turbulent fluids^44^. Notably, it has been shown that Lévy motion underlies the propagation of gamma (30–100 Hz) burst patterns in the MT area of marmoset monkeys^45^, and that hippocampal sharp wave ripples exhibit random movements with occasional long jumps^46^.

To quantify the Lévy motion of the localized activity pattern (Fig. 2a), we calculate its mean-squared displacement and the distribution of the increment of the pattern’s movement as in ref. 43. We track the pattern over time and calculate its center of mass (CoM), ${\hat{{{{{{{{\bf{s}}}}}}}}}}_{t}=({\hat{x}}_{t},{\hat{y}}_{t})$ (see “Methods”). As shown in Fig. 2a, the CoM trajectories exhibit random diffusive properties with variable step sizes, resulting in small-movement clusters occasionally interspersed by long jumps to new locations. The mean-squared displacement of the pattern can be calculated based on the CoM as, ${{{{{{{\rm{MSD}}}}}}}}({{\Delta }}t)=\langle \parallel {\hat{{{{{{{{\bf{s}}}}}}}}}}_{t+{{\Delta }}t}-{\hat{{{{{{{{\bf{s}}}}}}}}}}_{t}{\parallel }^{2}\rangle$, where Δ*t* is the time lag. As shown in Fig. 2b, the mean-squared displacement is linear on a log–log scale, indicating that it is a power function of Δ*t*, such that MSD(Δ*t*) ∝ Δ*t*^*η*^. The diffusion exponent *η* determines the type of the random motion: a value of *η* = 1 indicates Brownian motion, commonly used for implementing MCMC sampling^47^; a value of *η* > 1 indicates a superdiffusive process and *η* < 1 indicates a subdiffusive process. We find that the localized activity pattern emerging in our network has *η* = 1.18 (Fig. 2b), indicating that its movement is superdiffusive. We further examine the distribution of the increment ${{\Delta }}{\hat{x}}_{t}={\hat{x}}_{t+{{\Delta }}t}-{\hat{x}}_{t}$ of the pattern’s CoM trajectory by fixing the time interval Δ*t* = 15 ms and find that it exhibits a heavy tail. The increments can be fitted to a symmetric Lévy stable distribution with index 1 < *α* ≤ 2 (see “Methods”); using maximum likelihood, we find that *α* = 1.28 (Fig. 2c). The tail of this distribution asymptotically follows a power law $p({{\Delta }}\hat{x}) \sim|{{\Delta }}\hat{x}{|}^{-1-\alpha }$, where *α* is often referred to as the tail index. The heavy-tailed, power-law distribution of the increment and the corresponding superdiffusion are the characteristic features of Lévy motions^28,48^.

We next illustrate how the localized activity pattern (i.e., assemblies of neurons) with Lévy motion is able to implement FNS. To provide a conceptual understanding of the computational goal of FNS, consider the probabilistic generative process shown in Fig. 1c. A stimulus **x** (such as an image) is defined in terms of its latent feature **s** (such as its orientation and color) and a global contrast level *c*. For clarity, we denote the true value of the stimulus feature in a particular trial as s* to distinguish it from the generic latent variable **s**. The stimulus **x** evokes a sensory response **r** (spike counts). We assume that the sensory response depends on the latent feature in the form of a probabilistic population code; that is, the sensory response *r*_*i*_ of each neuron *i* follows an independent Poisson distribution with a bell-shaped firing rate profile centered at s* with height proportional to the stimulus contrast *c* (see Eq. (10) in “Methods”). Thus, the sensory response **r** conveys both information about the latent feature s* as well as the strength of the sensory evidence (proportional to stimulus contrast). The sensory response is used as the feedforward input to the recurrent circuit, where probabilistic inference is performed by combining the sensory evidence conveyed by the feedforward input and the prior embedded in the recurrent circuit. Mathematically, this is expressed with Bayes’ rule

1 $$p({{{{{{{\bf{s}}}}}}}}|{{{{{{{\bf{r}}}}}}}})=\frac{p({{{{{{{\bf{r}}}}}}}}|{{{{{{{\bf{s}}}}}}}})p({{{{{{{\bf{s}}}}}}}})}{p({{{{{{{\bf{r}}}}}}}})},$$

where *p*(**s**∣**r**) and *p*(**s**) represent the posterior and the prior distributions, respectively. In our FNS approach, a sample $\hat{{{{{{{{\bf{s}}}}}}}}}$ from the feature space is represented by the instantaneous CoM of the localized spiking pattern in the recurrent circuit. The distribution resulting from the random motion of the localized activity pattern then approximates the posterior distribution.

In this section, we first consider the simple case of sampling a unimodal distribution corresponding to sensory evidence with a flat prior to illustrate the key spatial and temporal properties of FNS. The neural circuit receives a unimodal feedforward input centered at the true value of the stimulus feature s*, with its strength proportional to the stimulus contrast level *c*, as described by Eq. (10) (“Methods”). Figure 2d shows a typical sample path traced by the CoM of the localized spiking pattern, which exhibits complex spatial and temporal dynamics. Spatially, the sampling path exhibits the signature of Lévy motion, i.e., the presence of occasional large jumps in space (Fig. 2d). Similar to the case of spontaneous activity, the distribution of the sample path increment is characterized by a heavy tail. Temporally, the sample path displays an oscillatory component as indicated by its autocorrelation (Fig. 2e), which can be fitted as a function of the form $\exp (-{{\Delta }}t/\tau )\cos (2\pi f{{\Delta }}t)$ with the decay constant *τ* ≈ 36 ms and the oscillation frequency *f* ≈ 5.5 Hz. The frequency of the oscillation is higher for the stronger contrast level (Fig. 2e). It is interesting to note that this frequency is in the range of theta oscillations (3–8 Hz), an elementary oscillatory component widely observed during environmental sampling tasks such as spatial attention sampling^30^ and whisking in rodents^49^. We will come back to this point in “Discussion”.

We next calculate the convergence speed of statistical estimates based on these samples toward the true value of the stimulus feature; in particular the convergence of the sample mean, ${\bar{{{{{{{{\bf{s}}}}}}}}}}_{T}=\frac{1}{T}\int\nolimits_{0}^{T}{\hat{{{{{{{{\bf{s}}}}}}}}}}_{t}dt$. As shown in Fig. 2, the mean-squared error of the sample mean, ${\mathbb{E}}[\parallel {\bar{{{{{{{{\bf{s}}}}}}}}}}_{T}-{{{{{{{{\bf{s}}}}}}}}}{*}{\parallel }^{2}]$, with s* denoting the true value of the stimulus feature, decreases to half-maximum at *T*_HM_ = 128 ms for contrast *c* = 1 and *T*_HM_ = 81 ms for *c* = 2, which are around the same order as the adaptation time constant *τ*^K^ = 80 ms and are several times the membrane time constant (*τ*_m_ = 15 ms). The rate of convergence is faster for the higher contrast level, as consistent with the observation that the frequency of oscillation increases with contrast (Fig. 2e). This result indicates that the activity pattern emerging from the circuit implements sampling-based representation of the stimulus features, and that the sampling process is quite efficient.

As the stimulus contrast level represents the strength of sensory evidence, the increase in contrast should lead to a reduction of the sample variance, as suggested by the generative model in Eq. (1) as well as by existing studies^13^. We find that this property is indeed satisfied in FNS: as shown in Fig. 2g, the sample variance $\frac{1}{T}\int\nolimits_{0}^{T}{({\hat{x}}_{t}-{\bar{x}}_{T})}^{2}dt$ (calculated over a time period *T* = 10 s) decreases with stimulus contrast. From the dynamics aspect, the reduction of sample variance with contrast is primarily due to the modulation of the random motion of the activity pattern by the feedforward input; that is, as the contrast level of the input increases, the CoM of the pattern becomes more concentrated around the center of the input.

### FNS with realistic neural response properties

**Fig. 3:**
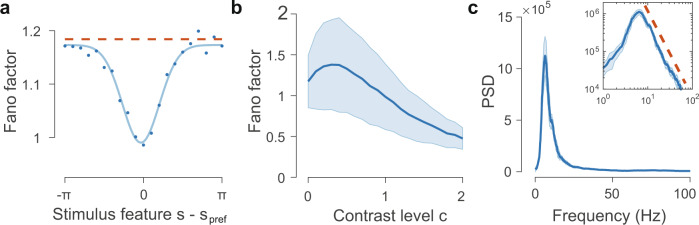
FNS accounts for neural response properties. **a** Fano factor tuning of neural responses for varying stimulus feature values *s* relative to the neuron's preferred stimulus *s*_pref_ for contrast level *c* = 1 (dots). Dashed line corresponds to spontaneous activity when *c* = 0. Fano factor is calculated using spike counts over 100 ms time windows. **b** Trough of the Fano factor in (**a**) as a function of the contrast level *c* of the feedforward input. Shades indicate the standard deviation across different neurons. **c** Theta oscillations revealed by the power spectral density (PSD) of the population firing rate with a peak frequency at 6.2 Hz. Solid line and shade indicate the average and standard deviation across 10 trials, respectively, each with a duration of 10 s. Inset: the PSD on a log–log scale reveals a power-law tail with an exponent of −2.28 (dashed line, offset for visibility).

We now demonstrate that the sampling dynamics of FNS capture key response properties of the cortex including the reduction of neural firing variability^3,29^. To this end, we calculate the spike-count Fano factor for individual neurons by using a fixed time window of Δ*t* = 100 ms. The Fano factor following stimulus onset has a pronounced drop compared to the Fano factor during spontaneous activity^3^. We find that this reduction of Fano factor displays a U-shaped dependence on the stimulus feature (orientation) as found in the middle temporal area of monkeys^29^, with the largest reduction occurring when the stimulus orientation is equal to the neuron’s preferred orientation (Fig. 3a). As shown in Fig. 3b, the Fano factor is greater than one for the spontaneous activity (*c* = 0) and is reduced by an amount largely proportional to stimulus contrast, with the exception of low contrast levels (*c* ≤ 0.3).

We also investigate how the Fano factor in the recurrent circuit depends on the width of the feedforward input. As shown in Fig. S1a, when the width of the input is smaller than that of the receptive field, the Fano factor decreases with the input width; the converse is true when the input width is larger than the receptive field. It has been shown that in the primary visual cortex of monkeys, Fano factor exhibits a similar trend as the stimulus size varies (see Fig. 2f of ref. 50). In addition, we find that as the input width increases, the uncertainty of the sampled distribution changes in the similar way as the Fano factor (Fig. S1b). This correspondence between the uncertainty of neural responses and that of the sampled distribution is due to the reduction (or increase) in the variability of the instantaneous firing rates of neurons as the variance of the samples (represented by the CoM of the spiking pattern) decreases (or increases).

Another key feature of neural responses arising from the sampling dynamics is that neural population firing rate possesses a theta oscillatory component (3–8 Hz), as indicated in its power spectral density (PSD) (Fig. 3c). Interestingly, these oscillations ride on top of a 1/f arrhythmic component. Certain oscillatory components such as theta accompanied by 1/f-like activity have been widely observed in the cortex^20,21^; they are particularly relevant for cognitive functions such as visual-spatial attention^51,52^. These results provide further neurophysiological validity of our circuit model of FNS.

### Circuit mechanism of FNS

We next elucidate the circuit mechanism underlying the emergence of the sampling dynamics in our circuit model. More specifically, we aim to pinpoint the origin of the two key features of FNS: the fractional Lévy motion and the theta oscillatory component.

**Fig. 4:**
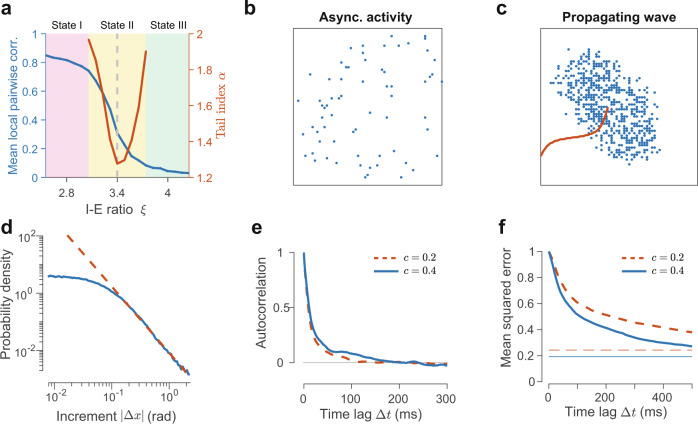
Neural circuit mechanism of FNS. **a** Phase diagram of the emergent activity states in the spiking neural circuit as the inhibition-to-excitation ratio *ξ* varies. Neural correlations (blue line) of spike counts reveal a state transition from the asynchronous state (State III, weak correlation) to propagating wave state (State I, strong correlation). Only within the transition regime (State II), the increment distribution of the trajectory of the localized spiking pattern follows the *α*-stable distribution with its power-law tail index *α* smaller than 2, indicating the presence of fractional Lévy motion. **b**, **c** Snapshots of the spiking pattern for State I and State II featuring asynchronous activity (*ξ* = 4.25, 15 ms time window) and a localized propagating wave (*ξ* = 2.55, 2 ms time window), respectively. Red curve indicates the CoM trajectory of the propagating wave. **d** When adaptation is removed, the histogram of the sample increments (solid line) still exhibits a power-law tail (dashed line). **e** Sample path autocorrelation shows the absence of anticorrelation as a result of removing neural adaptation. **f** Mean squared error of the sample mean decreases slowly with the time lag, with a baseline due to quenched noise (randomness in synaptic connectivity).

The circuit model exhibits a rich repertoire of dynamical activity states, ranging from the asynchronous to propagating wave states^40^. Particularly, by varying the I–E ratio (*ξ*), it has shown that around the transition state between the asynchronous (i.e., disordered) and localized propagating wave (i.e., ordered or coherent) states, the circuit model explains a range of nonlinear neural response properties; this state transition has been characterized by calculating the susceptibility and the branching indices of neural spikes^40^. Figure 4a shows that when the I–E ratio *ξ* is large, the network exhibits an asynchronous state without any structured patterns (State III, Fig. 4b), in which neural correlations (see “Methods”) are low. On the other hand, when the I–E ratio is small, coherent patterns emerge from the circuit in the form of a localized propagating wave; this wave pattern propagates across the neural circuit with a relatively smooth and regular trajectory (Fig. 4c). In this state (State I), neural correlations are much greater than those in the asynchronous state. In the transition regime (State II) between these two states (State I and State III), neural correlation changes rapidly as *ξ* varies. Only in State II, the CoM of the pattern exhibits fractional Lévy motion with small steps occasionally interrupted by long jumps (Fig. 1a)^51^, as indicated by a tail index of *α* < 2 with the trough of *α* coinciding with the transition point at *ξ* = *ξ*_*c*_ = 3.4. This value of I–E ratio (*ξ*_*c*_ = 3.4) is quantitatively consistent with that measured in the visual cortex of awake mice^53^. It is interesting to note that this dynamical mechanism underlying Lévy motion in our circuit model is similar to other complex physical systems whose critical phase transitions are essential for the emergence of Lévy motion^54^.

Previous studies have shown that negative feedback mechanisms such as spike-frequency adaptation or depression are essential for the emergence of oscillations^55,56^. Our realistic spiking network model also incorporates spike-frequency adaptation in the form of slow potassium currents, which could be the origin of oscillation in our model. To test this, we remove the adaptation completely from the network by setting the potassium current in Eq. (5) to zero. We use the spontaneous activity to re-calibrate the inhibitory synaptic strength by a factor of 1.2 so that the neural dynamics is restored to the transition regime between the asynchronous and localized pattern states. Within this regime, the increments of the CoM of the localized activity pattern still follow a symmetric Lévy stable distribution, with the tail index *α* = 1.19 (Fig. 4d), indicating that the pattern moves in a fractional Lévy manner. However, we find that in the circuit without adaptation, the sample autocorrelation function of the sample path decays exponentially as $\exp (-{{\Delta }}t/\tau )$ with *τ* = 13–19 ms when the pattern samples the unimodal distribution; it does not show any feature of oscillation or anticorrelation (Fig. 4e). This lack of theta oscillations in the circuit model without adaptation results in a slower decay (time-at-half-maximum Δ*t*_HM_ = 317 ms for *c* = 1 and Δ*t*_HM_ = 220 ms for *c* = 2) of the mean squared error of the mean estimate (Fig. 4f) than for the case with oscillations (Fig. 2f), indicating that theta oscillations play a role of speeding up sampling of FNS. These results indicate that the oscillatory component of FNS originates from neural adaptation in our model.

### A mathematical model of fractional neural sampling

We next develop a mathematical model to gain further theoretical insights into the probabilistic sampling processes implemented in our spiking circuit model. Based on this mathematical model, we reveal the unique computational properties of FNS through highlighting the functional roles of temporal oscillations and Lévy motion.

As demonstrated above, the spiking pattern behaves like a random walker that exhibits far richer spatiotemporal dynamics than Brownian motion: the pattern exhibits occasional long jumps in space, a characteristic feature of Lévy motion, and an oscillatory component in its CoM. To model a random walk with these features, we use a stochastic differential equation (SDE) driven by Lévy motion with an auxiliary momentum term

2 $$d{\hat{x}}_{t} 	=\gamma b({\hat{x}}_{t})dt+\beta {\hat{v}}_{t}dt+{\gamma }^{1/\alpha }d{L}_{t}^{\alpha },\\ d{\hat{v}}_{t} 	=\beta b({\hat{x}}_{t})dt,$$

where ${\hat{x}}_{t}$ is the CoM of the activity pattern, *v*_*t*_ is an auxiliary variable representing momentum, *β* is the damping coefficient, *b*(*x*) is a drift term related to the probability landscape, *γ* is the strength of the noise, and ${L}_{t}^{\alpha }$ is the Lévy motion whose step sizes over a time period Δ*t* follow a symmetric Lévy stable distribution ${{{{{{{\mathcal{S}}}}}}}}\alpha {{{{{{{\mathcal{S}}}}}}}}(\alpha,{{{\Delta }}t}^{\frac{1}{\alpha }})$ possessing a power-law tail with a tail index 1 < *α* ≤ 2 (see “Methods”)^28^. It has been shown that the momentum term *v*_*t*_ is responsible for generating temporal oscillations in the trajectory of the random walker^57^, with the frequency of the oscillations controlled by the damping coefficient *β*. Thus, the mathematical model (Eq. (2)) is able to capture the essential dynamical features of the localized pattern, i.e., the Lévy motion and the temporal oscillations. For analytical tractability we consider the case with one spatial dimension.

As the trajectory of a random walker evolves over time, its probability density converges towards a stationary distribution (target distribution) *π*(*x*). Therefore, the trajectory of the random walker, after a sufficient burn-in time, can be considered as approximate samples from *π*(*x*). In the case of Eq. (2), the stationary distribution *π*(*x*) is the stationary solution (marginalized by treating momentum *v* as a nuisance variable) to the corresponding fractional Fokker-Planck equation, and it is related to the drift term *b*(*x*) by (see Method for mathematical derivations of this result)

3 $$b(x)=\frac{{{{{{{{{\mathcal{D}}}}}}}}}_{x}^{\alpha -2}[\pi (x){\partial }_{x}\log \pi (x)]}{\pi (x)},$$

where ${{{{{{{{\mathcal{D}}}}}}}}}_{x}^{\alpha -2}$ is the partial Riesz fractional derivative. Using Eq. (3), we can determine the drift term *b*(*x*) required for producing samples from any desired target distribution *π*(*x*).

This mathematical model provides a simple yet effective theoretical framework for revealing the key computational properties of FNS. Without loss of generality, we set the tail index in Eq. (2) to be *α* = 1.2, similar to the tail index characterizing the Lévy motion of the spiking patterns emerging in the neural circuit model, and set *β* = 1 to capture the oscillatory aspect of FNS; other values of 1 < *α* < 2 and *β* > 0 would generate qualitatively similar results. We then compare the computational performance of this default model with three other sampling processes: (1) Sampling without oscillation (*β* = 0) but with fractional Lévy motion (*α* = 1.2). (2) Sampling with oscillation (*β* = 1) but without fractional Lévy motion (*α* = 2), which is the standard Hamiltonian Monte Carlo (HMC) sampling^57^. Such Hamiltonian dynamics have been previously mapped to a neural network with excitatory and inhibitory populations^58^ for performing efficient sampling. (3) Sampling with neither oscillation nor fractional Lévy motion (*α* = 2 and *β* = 0). For this case, the sampling process reduces to the standard MCMC driven by Brownian motion^47^, called Langevin sampling, which is used as a general-purpose algorithm in machine learning; it has been proposed that neural networks may implement such sampling^14–16,59^.

**Fig. 5:**
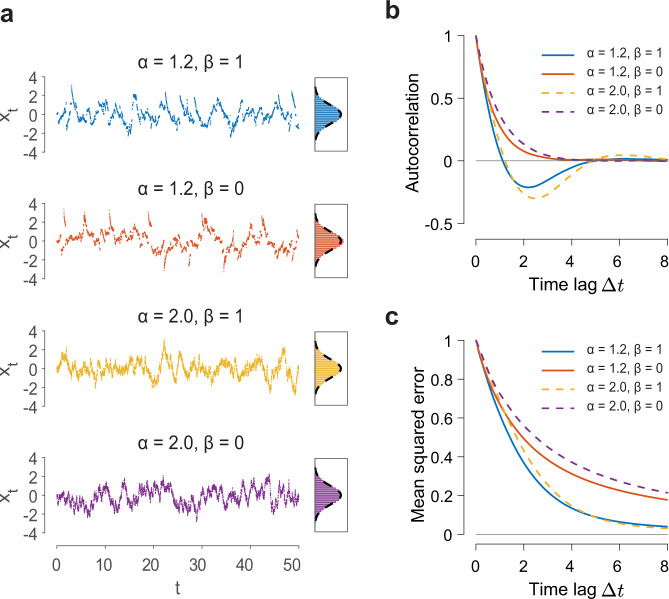
Properties of different sampling approaches. **a** Sampling processes driven by fractional Lévy motion (*α* = 1.2, top two panels) exhibit long jumps, in contrast to their non-fractional counterparts (*α* = 2, bottom two panels). Right panels: sample histograms agree with the target distributions (dashed line). **b** Autocorrelation functions of different sampling processes. The processes with momentum (*β* = 1) exhibit an oscillatory component. **c** Mean squared error as a function of Δ*t*. The two cases with an oscillatory component (*β* = 1) have significantly faster convergence to the true mean (gray line) than those without (*β* = 0). Fractional Lévy dynamics do not affect the convergence speed in a significant way.

Typical sample paths of the four cases for sampling a standard normal distribution with zero mean and unit variance are shown in Fig. 5a, each exhibiting distinct features. Immediately noticeable are the jumps in the sample paths of the two sampling processes powered by the fractional Lévy motions, but are absent in their non-fractional counterparts. Despite the drastic differences in the sample path structure, all four cases are able to produce samples from the target distribution, as seen through the agreement between the sample histogram and the target distribution (right panels in Fig. 5a). The finer temporal structures of these sampling processes are further revealed through calculating the autocorrelation function of the sample path, $\langle {\hat{x}}_{t+{{\Delta }}t}{\hat{x}}_{t}\rangle$, as we have calculated for the spiking pattern in the circuit model. As shown in Fig. 5b, the autocorrelation functions of the two cases without the oscillatory component (*β* = 0) decay exponentially to zero. However, those with the oscillatory component (*β* = 1) drop to zero quickly and exhibit negative-going lobes. This indicates that successive samples rapidly decorrelate, speeding up the sampling processes and thus resulting in a faster convergence to the target distribution. As shown in Fig. 5c, the mean-squared error decays significantly faster for the two cases with the oscillatory component (*β* = 1), with a time-at-half-maximum of 1.42 for *α* = 1.2 and 1.70 for *α* = 2, than that without oscillation (*β* = 0), with a time-at-half-maximum of 1.95 for *α* = 1.2 and 2.51 for *α* = 2. These results are consistent to the observations in the spiking neural circuit model in Fig. 2, and show that the temporal oscillatory property of FNS plays an essential role in improving sampling speed. Note that the cases with the momentum (*β* > 0) and without the momentum (*β* = 0) in the mathematical model correspond to neural sampling in the circuit model with and without adaptation, respectively. In the mathematical model, the momentum pushes successive samples further apart, giving rise to the oscillatory behavior and accelerating the sampling speed. In the neural circuit model, adaptation plays a similar role by pushing the localized activity pattern away from its present location, thus speeding up sampling.

We next elucidate that FNS possesses the powerful computational property of sampling multimodal distributions with far-away modes. For this purpose, we set the target distribution as in Eq. (3) to be a bimodal Gaussian mixture

4 $$\pi (x)=\frac{1}{2}\frac{1}{\sqrt{2\pi }\sigma }\exp \left[-\frac{{(x-{s}{*})}^{2}}{2{\sigma }^{2}}\right]+\frac{1}{2}\frac{1}{\sqrt{2\pi }\sigma }\exp \left[-\frac{{(x+{s}{*})}^{2}}{2{\sigma }^{2}}\right].$$

**Fig. 6:**
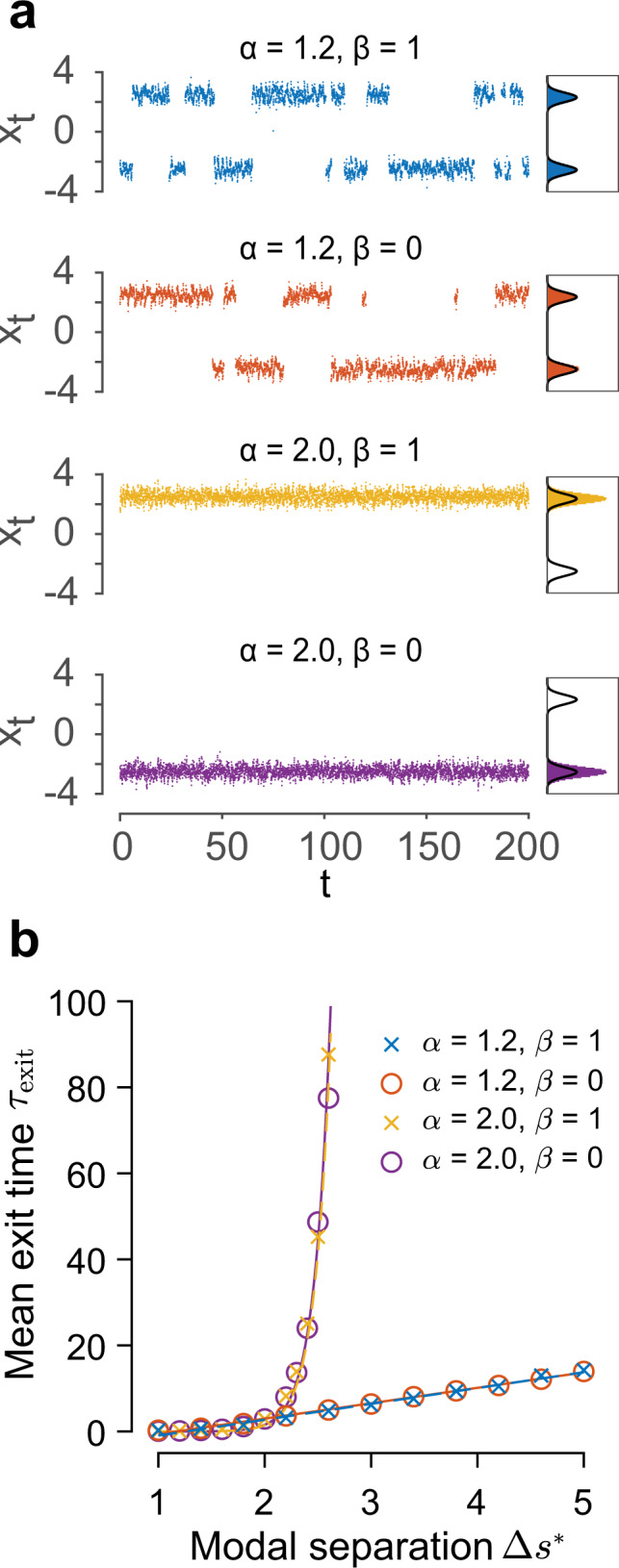
Illustration of the computation properties of FNS for sampling bimodal distributions. **a** Sampling processes driven by fractional Lévy motion (*α* = 1.2, top two panels) are able to alternate between two modes rapidly (modal separation Δs* = 5), rather than being stuck at one mode as their non-fractional counterparts (*α* = 2.0, bottom two panels). Right panels: sample histograms and target distribution (solid line). **b** Mean exit time of the sampling processes driven by fractional Lévy motion grows linearly with modal separation, whereas those driven by Brownian motion grow exponentially; averaged across 24 trials of duration *T* = 10^4^. Circles and crosses represent results from the mathematical model; solid lines denote the fitted exponential and linear curves.

Without loss of generality, we set s* = 2.5 and *σ* = 0.32. For clarity and simplicity, here we consider a bimodal distribution, but the results can be generalized to any multimodal distributions. Note that the modal separation Δs* is significantly larger than the modal width *σ*, implying vanishingly small probability density between the modes; in other words, the modes are separated by a high potential barrier, $U(x)=-\log \pi (x)$, which is extremely difficult to penetrate. For the two fractional cases (*α* = 1.2), despite the impenetrable energy barrier between the two modes located at ± s*, the sample path is able to switch intermittently between them due to the inherent long jumps of Lévy motion (Fig. 6a). This property enables efficient sampling of the bimodal distribution, with the sample histogram matching the target distribution. In contrast, in both sampling processes driven by conventional Brownian motion (*α* = 2), i.e., the Langevin Monte Carlo sampling and the Hamiltonian Monte Carlo (HMC) sampling, the sample paths are completely trapped in one of the modes and are unable to explore the entire state space, thus failing to sample bimodal distributions. Particularly, we stress that while HMC has been previously implemented in neural networks for speeding up sampling processes, it cannot on its own resolve the issue of sampling multimodal distributions with far-apart modes because the sampler has a negligible chance to gather a sufficiently large momentum to overcome high potential barriers^60^.

The Lévy motion can fundamentally improve the mixing ability of the sampling process, that is, the ability to traverse through the probability landscape including the low-probability regions between the two modes. To quantify this mixing property, we calculate the exit time *τ*_exit_ (see “Methods”), which is the duration the random walker remains in one of the modes, and examine how the mean exit time changes with the modal separation Δs*. As shown in Fig. 6b, the dependence of the mean exit time on the modal separation has striking differences between the cases with and without fractional Lévy dynamics. For the two cases driven by fractional Lévy motions, the mean exit time increases linearly with the modal separation; in contrast, for the conventional cases driven by Brownian motion (Langevin sampling and Hamiltonian sampling), either with or without the oscillatory component, the mean exit time blows up exponentially as Δs* increases. Note that these results are achieved with all the other parameters such as the strength of the noise and the shape of the probability landscape fixed. Therefore, the mixing ability can be fundamentally attributed to the fractional Lévy motion ${L}_{t}^{\alpha }$. The linear dependence of the mean exit time on modal separation is the hallmark of FNS, indicating that it can effectively sample multimodal distributions regardless of variations in modal separation.

### Relations between the drift term and neural circuit parameters

As illustrated in the mathematical model of FNS, the drift term *b*(*x*) determines the sampled distribution according to Eq. (3). To demonstrate that the spiking neural circuit performs non-trivial probabilistic computations as in the mathematical model, it is necessary to show how the drift term is related to the feedforward input (for encoding sensory evidence) and recurrent synaptic connections (for encoding prior). Due to the mathematical intractability of the spiking neural circuit model, we employ a neural field model (Eq. (27)) to derive the relationships between *b*(*x*) and parameters of the feedforward input and the recurrent synaptic weights (see “Methods” for the neural field model and Supplementary Information for details of the mathematical derivations); we then numerically validate these relationships in our spiking neural circuit model, thus providing insights into the probabilistic computations implemented in the spiking neural circuit.

**Fig. 7:**
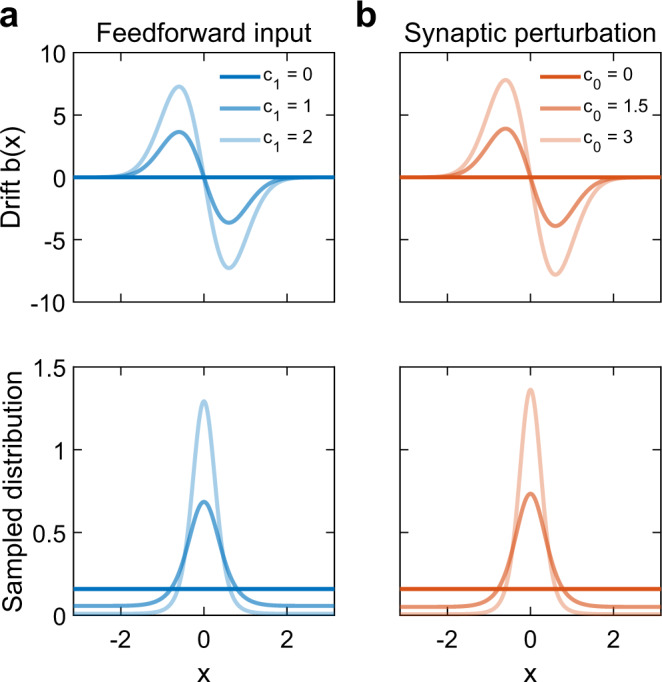
Drift term related to feedforward input and synaptic perturbations. **a** Drift term (upper panel) and sampled distribution (lower panel) when the neural field model receives a feedforward input with varying contrast levels *c*_1_. **b** Drift term (upper panel) and sampled distribution (lower panel) when the neural field model receives synaptic weight perturbation with varying strengths *c*_0_.

Figure 7a (upper panel) shows *b*(*x*) when the neural field model receives a bell-shaped feedforward input (Eq. S5) centered at *s*_1_ = 0 with varying contrast levels *c*_1_. The drift is positive when *x* < *s*_1_ = 0 and is negative when *x* > *s*_1_ = 0, indicating that the localized pattern experiences a drift toward *s*_1_ = 0 and subsequently samples from a probability distribution centered at *s*_1_. Further analysis shows that the drift magnitude is proportional to stimulus contrast *c*_1_ (Eq. (28)). Using *b*(*x*), we then analytically obtain an explicit solution to the resulting sampled distribution (Fig. 7a, lower panel), whose variance is inversely proportional to *c*_1_ (Eq. S20). In addition, our analysis shows that a bell-shaped perturbation centered at *s*_0_ = 0 to the recurrent synaptic weights (Eq. S6) can induce a drift *b*(*x*) with a similar shape around *s*_0_ (Fig. 7b, upper panel); the magnitude of the drift is proportional to the strength of synaptic perturbation *c*_0_ (Eq. (28)). For this case, we also derive the corresponding sampled probability distribution (Fig. 7b, lower panel) and find that its variance is inversely proportional to *c*_0_ (Eq. S21); note that this probability distribution caused by changes in synaptic weights is the prior distribution.

We next calculate the drift *b*(*x*) in the spiking neural circuit model from the CoM trajectory of the spiking pattern. Since the increments of the CoM are equal to the sum of a deterministic drift and random noise according to the mathematical model (Eq. (2)), we can obtain *b*(*x*) by averaging the increments of the CoM trajectory over time and across trials to remove the noise. We then divide the two-dimensional space into bins and calculate the drift as a function of the feature coordinate *x*. We find that when the neural circuit receives a feedforward input as in Eq. (10), the drift *b*(*x*) is positive for *x* < *s*_1_ = 0 and negative for *x* > *s*_1_ = 0, resulting in a sampled distribution centered at *s*_1_ (Fig. S2a). A bell-shaped perturbation to the recurrent synaptic weights (Eq. (12)) also yields a similar outcome (Fig. S2b), thus properly embedding the prior to the circuit. These results are qualitatively consistent with the analytical results based on the neural field model, indicating that the drift term is determined by the feedforward input and recurrent synaptic connectivity.

### Fractional neural sampling of multimodal distributions

We next validate the powerful mixing property for sampling multimodal distributions in our spiking circuit implementation of FNS. For this purpose, we change the spatial profile of the feedforward input to be the superposition of two Gaussian functions centered at **s**_1_ and **s**_2_ with contrast levels *c*_1_ and *c*_2_, respectively (Eq. (11) in “Methods”); for simplicity, here we use a bimodal input as an example, but multimodal distributions can be similarly sampled in the circuit. Such multimodal inputs can be thought as representing sensory responses from multiple sensory channels, as in multisensory perception tasks^61^. It may also arise from a single stimulus with alternative interpretations such as the spatial orientation of a Necker cube. In both cases, the true state s* of the latent stimulus feature could be either **s**_1_ or **s**_2_.

**Fig. 8:**
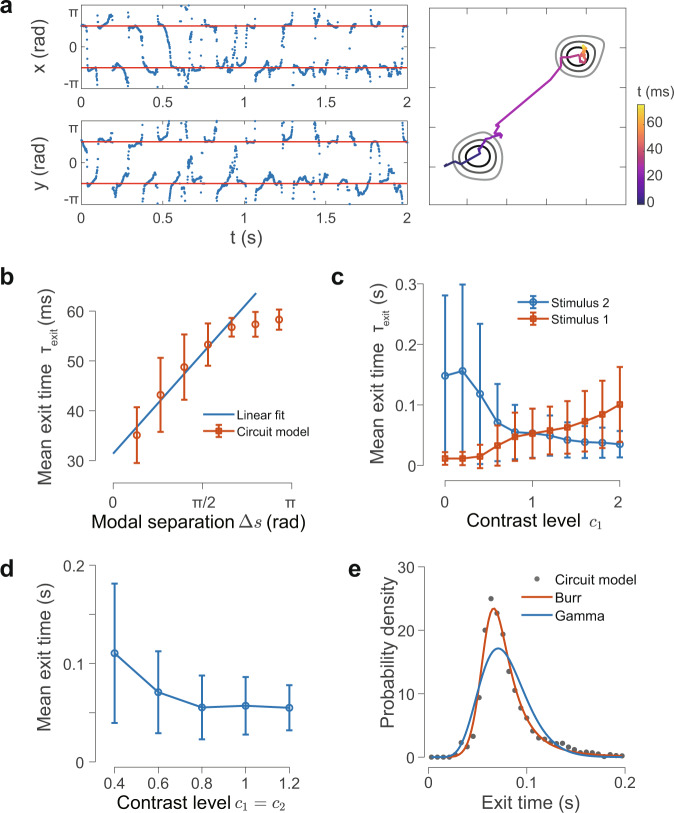
Circuit implementation of FNS for sampling bimodal distributions. **a** Time series of the *x*- and *y*-coordinates of the sample path when *c*_1_ = *c*_2_ = 1. Samples (blue dots) are concentrated around the stimuli (red solid line) and switch rapidly between them without being trapped. Right panel: contour plot of the log sample probability density with a segment of the sample path overlaid on top. **b** Mean exit time (circle) grows linearly with the modal separation (with saturation due to periodic boundary conditions). **c** Mean exit time for varying *c*_1_ with *c*_2_ fixed (*c*_2_ = 1). **d** Mean exit time decreases as both contrast levels are kept the same and increased simultaneously. Error bars in **b**–**d** show the standard deviation of the exit time within trials. **e** Exit time (perceptual dominance duration) follows a right-skewed distribution better fitted with a Burr distribution (red line) with a more slowly decaying tail than a gamma distribution (blue line).

Figure 8a shows that the localized pattern wanders around the location of one of the modes for a short while and then jumps to the other one, thus alternately sampling these modes in a similar fashion as in the mathematical model. Consequently, the sampling process leads to bimodal sample histograms whose modes are separated by low-probability regions (right panel in Fig. 8a). To calculate the exit time for the localized activity pattern performing sampling, we assign the spatial coordinates of the CoM trajectory at each time moment to one of the stimulus peaks depending on which peak location it is closer to. The exit time is then defined as the duration the trajectory spends in one region before it switches to the other. We investigate how the exit time changes as the modal separation Δ*s* increases. As shown in Fig. 8b, the mean exit time ranges from 30 to 60 ms, indicating that the alternating sampling between the two modes occurs rapidly. The mean exit time increases linearly for ${{\Delta }}s \, < \, \frac{3}{4}\pi$, before it saturates due to the periodic boundary condition of the circuit model. This result indicates that FNS implemented in the neural circuit exhibits the salient feature as predicted in the mathematical model; that is, the mean exit time of the activity pattern increases linearly with the modal separation.

We next demonstrate the effectiveness of our circuit implementation of FNS for different contrast levels by systematically varying *c*_1_ while keeping *c*_2_ = 1 fixed. Rather than being trapped in the spot with a higher contrast level, the activity pattern is still able to dynamically switch between the two modes intermittently. By calculating the exit time, we find that when the contrast level of one of the stimuli becomes stronger, the mean exit time from this stimulus location increases monotonically while that from the other stimulus location decreases (Fig. 8c).

In summary, these results indicate that FNS can sample multimodal probability distributions in such a flexible and effective way that it is insensitive to their modal separations and contrast differences, thus retaining the excellent mixing ability. These dependencies of the exit time on modal separations and contrast levels form testable predictions of our FNS theory.

### Sampling dynamics of FNS underlie perceptual switching

When presented with a stimulus with alternative interpretations such as the Necker cube or conflicting stimuli such as in binocular rivalry, humans may experience two alternating percepts^31^. Such phenomenon known as perceptual switching can be naturally explained in the framework of FNS. To model a simple case of binocular rivalry in which two conflicting stimuli with opponent colors are presented to two eyes, we add inputs to two neural groups whose preferred hues differ by *π*, as in existing modeling studies^62^. Alternations between the responses of the two neural groups (left panel, Fig. 8a) are interpreted as switching between different percepts. The exit time from each neural group calculated above is equivalent to the dominance duration of each percept in the context of perceptual switching. The result with varying contrast level *c*_1_ and fixed *c*_2_ (*c*_2_ = 1), as shown in Fig. 8c, is thus consistent with one of the key properties of perceptual switching of binocular rivalry^31^. When the contrast level of one stimulus is increased from zero with the other fixed, it primarily decreases the mean dominance duration of the stronger percept while increasing that of the weaker one to a lesser extent and equi-dominance is reached when the contrast levels of both stimuli are equal (*c*_1_ = *c*_2_). Further increasing the contrast level of the stimulus primarily increases the dominance duration of the stronger percept while decreasing that of the weaker one to a lesser extent^31^. We also increase the contrast levels of the two stimuli simultaneously and find that the mean dominance duration of both percepts decreases (Fig. 8d), which is another key property of perceptual switching^31^.

In addition, dominance durations in our model follow a right-skewed distribution (Fig. 8e), consistent with psychophysical studies of perceptual switching. Although the specific forms of this type of right-skewed distribution have been found to vary across different experimental and modeling studies, which are still a matter of debate^31^, we find that the dominance durations in our model are best fitted by a right-skewed distribution called Burr distribution rather than a gamma distribution (see “Methods” for the fitted parameters of these distributions). The key difference is that the former features a slowly decaying power-law tail whereas the latter has a faster decaying exponential tail. It is interesting to note that such a slowly decaying tail in the distribution of perceptual dominance duration has been previously found in experiments^63^, albeit not explicitly stated. Our results thus propose another right-skewed distribution for capturing dominance duration, which can be tested in future experimental studies.

As in our model, previous noise-driven attractor models have successfully reproduced the dependence of perceptual switching on stimulus contrast and the distribution of dominance duration through a combination of adaptation and externally added noise^64^. In our model, however, intrinsically emergent fluctuations in the form of fractional Lévy motion are the dynamical origin of perceptual switching. To demonstrate this, in the transition regime of the circuit model without adaption as described above (see Fig. 4), we apply the same feedforward input described by Eq. (11) and find that the localized activity pattern exhibits rapid switching between the two neural groups (Fig. S3). This result thus indicates that the fractional Lévy motion underlies perceptual switching. It is important to note that in our model, the movement of the activity pattern in space has large fluctuation across multiple scales, as evidenced by the heavy-tailed, power-law distribution of its movement step sizes; however, switching behaviors between different attractors possess characteristic scales in existing modeling studies. In ref. 15, it has been shown that noise-driven activity states in a neural network model can be used to perform MCMC sampling and to model perceptual switching. In this study, the effect of modal separation on perceptual switching was not investigated; however, as demonstrated in the mathematical model (Eq. (2)), at the algorithmic level, the MCMC sampler with Langevin dynamics is unable to traverse through low-probability regions, particularly when multimodal distributions have far-apart modes. In contrast, the FNS theory suggests that Lévy motion with large fluctuations emerging from the spiking neural circuit can serve as a robust mechanism for explaining perceptual switching, as the FNS-based sampling dynamics are insensitive to modal separation.

### FNS-based perceptual inference

We next illustrate the properties of FNS-based probabilistic inference particularly through accounting for the key features of human perceptual inference in a motion direction task^32^. In this experiment, subjects were presented with visual stimuli consisting of moving dots, some of which move in the same direction while the rest move in random directions; the fraction of dots moving in the same direction is called coherence. It has been shown that the mean and variance of perceptual estimates match those predicted by the basic Bayesian observer model. However, the distribution of the perceptual estimates was found to be bimodal^32^; this observation was at odds with the basic Bayesian observer model which gave rise to a unimodal posterior. We now demonstrate that FNS-based inference provides a neural mechanism for resolving this puzzle and further makes new predictions about perceptual inference.

To proceed, we first establish a connection between the experimental setup and our model by interpreting the *x*-component of the 2D feature space as motion direction. The *x*-component of the CoM of the localized pattern at a particular time instant thus represents the perceived motion direction, whereas the *y*-component is considered as representing a nuisance parameter. We assume that the visual stimulus is represented through a population code as a bell-shaped feedforward input to the spiking neural circuit model (Eq. (10)), such that its center **s**_1_ represents the motion direction and its contrast *c*_1_ is proportional to the motion coherence, similar to that used in ref. 32. With this understanding, we compare the FNS-based inference results with the experimental results by carrying out the similar procedures as in the original experiment. In the experiment, the subjects were first trained with a block of visual stimuli to learn a prior distribution, and then were presented with a visual stimulus for a brief period of 300 ms before the subject was required to report the motion direction. Correspondingly, in our spiking neural circuit model we first embed a prior by applying a bell-shaped perturbation to the synaptic weights centered at **s**_0_ with amplitude *c*_0_ according to Eqs. (12) and (13) (see “Methods”). As illustrated above, these synaptic changes can be used to properly embed a prior to the recurrent circuit (Fig. 7). We then add a unimodal feedforward input with contrast *c*_1_ centered at **s**_1_ (Eq. (10)), which encodes the sensory evidence about the motion direction of the visual stimulus. The sampler (i.e., the localized activity pattern) is able to switch freely between the prior and the newly added sensory evidence within the short stimulus presentation period. The instantaneous position of the activity pattern at the end of the stimulus presentation is then interpreted as the perceptual estimate.

**Fig. 9:**
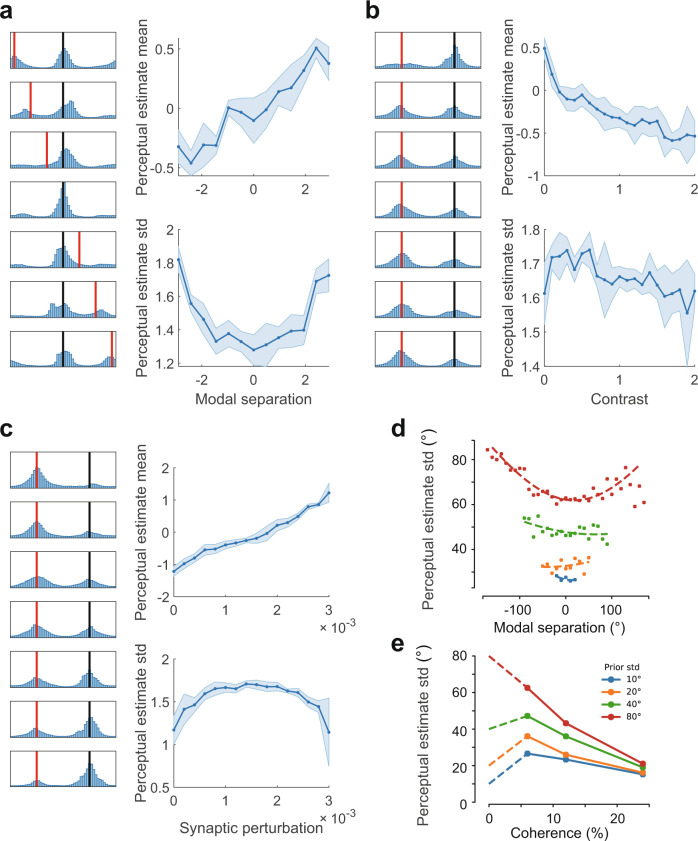
FNS-based perceptual inference. Perceptual estimate histogram, mean and standard deviation for: **a** varying modal separations between the stimulus (red bar) and the prior (black bar); **b** for varying stimulus contrast and fixed prior; **c** for varying prior strength (synaptic perturbation) and fixed stimulus contrast. Shades indicate the standard deviation across 20 trials. **d**, **e** Statistics of estimate distribution based on reanalysis of published experimental data. **d** Dependence of perceptual estimate variance (dots) on the modal separation can be approximately fitted by quadratic functions (solid curves), excluding outliers due to periodic boundary conditions. Stimulus coherence is fixed at 6%. **e** Standard deviation of the perceptual estimates decreases with stimulus coherence (solid lines), except for low coherence levels. Dashed lines denote extrapolations based on the theoretical argument that the perceptual estimate distribution should be equal to the prior when the coherence is zero. Modal separation is fixed at 20°.

We then use our model to explain the three sets of experiments in ref. 32. The first set of experiments involves fixing the coherence of the random moving dots and the prior while varying the motion direction relative to the prior mean. Correspondingly, in our neural circuit model, we fix the contrast level *c*_1_ = 0.5 in Eq. (10) and the strength of synaptic perturbation *c*_0_ = 0.002. Without loss of generality, we fix the prior mean **s**_0_ = (0, 0) and systematically vary the center **s**_1_ = (*s*, 0) of the feedforward input with *s* varying from −*π* to *π*. As shown in Fig. 9a (left panel), the estimate distribution is characterized by two peaks: one corresponding to the prior centered at the origin and another corresponding to the sensory evidence with varying motion directions. Notably, the similar feature of bimodality versus modal separation has been observed in the experiments^32^ (see Fig. 4 of their paper). As illustrated above, the bimodality in our circuit model arises as the spiking activity pattern intermittently alternates between the two modes (Fig. 8a). We further quantify the FNS-based probabilistic inference by calculating the mean and variance of perceptual estimates across trials. As the center of the feedforward input (thus the modal separation relative to the prior at the origin) increases from −*π* to *π*, the mean of the bimodal perceptual estimate distribution also increases from negative to positive values in an approximately linear fashion (Fig. 9a, top right panel); this relationship is consistent with the experimental results^32^ (see Fig. 3a of their paper). The variance of the perceptual estimate is approximately a quadratic function of modal separation (Fig. 9a, bottom right panel). We find that the linear and quadratic dependences of the mean and variance of perceptual estimates on the modal separation are also consistent with the analytical results obtained from the neural field model (see Eqs. S23–S24 in Supplementary Information).

To test the predictions from our inference results, we re-analyze the published data^32^ and find that the variance of the perceptual estimates also matches a quadratic curve when the prior standard deviation is large (80 degrees), as shown in Fig. 9d (red dots). However, for smaller prior standard deviations (Fig. 9d, green and orange dots), whether the experimental data matches our prediction remains inconclusive due to the limited data range^32^. Specifically, since the perceptual stimuli used in the experiments were pooled from the same dataset with which the prior was trained, its range was limited by the prior standard deviation (e.g., for a prior std of 10 degrees, the range of the presented stimulus is only about ±20 degrees). Future experiments could test our predictions by extending the range of the perceptual stimuli even when the prior standard deviation is small.

The second set of experiments involves varying the motion coherence while fixing the prior uncertainty and the distance of motion direction relative to the prior mean^32^. In our neural circuit model, this corresponds to varying the contrast level of the feedforward input *c*_1_ while fixing the strength of the synaptic perturbation *c*_0_ = 0.002 and the modal separation between them. The sample distributions for this case are shown in Fig. 9b (left panel), likewise exhibiting bimodality. As the contrast level increases, the height of the mode corresponding to sensory evidence increases whereas the height of the prior decreases. The mean and variance of perceptual estimates are also largely consistent with the experimental results: as the contrast level of the stimulus increases from zero, the mean of the bimodal perceptual estimate distribution gradually shifts from the prior at **s**_0_ toward the sensory evidence at **s**_1_ (Fig. 9b, top right panel). Meanwhile, the perceptual estimate variance decreases with the contrast level, but with the exception at low contrast levels (Fig. 9b, bottom right panel).

This finding that the variance of perceptual estimates varies non-monotonically with the stimulus coherence therefore provides a testable prediction for future experimental studies. By re-analyzing the published data^32^, we find that the perceptual estimate standard deviation decreases with the stimulus coherence for a number of coherence values (Fig. 9e). Experimental data for coherence smaller than 5% is unavailable so whether monotonicity is violated cannot be directly verified. However, if we impose the theoretical argument that the perceptual estimates should simply be samples from the prior distribution when the coherence is zero, we can then extrapolate the estimate standard deviation with the prior standard deviation. By presenting all data points together, we find that the monotonicity is indeed violated (Fig. 9e). To directly test our prediction, future experiments should more closely examine changes in the variance of perceptual estimates when the stimulus contrast levels are low.

The third set of experiments involves varying the uncertainty of the prior while fixing the coherence of the stimulus and the motion direction^32^. In the neural circuit model, this corresponds to varying the strength of the heterogeneous synaptic perturbation *c*_0_ while fixing the contrast of the feedforward input *c*_1_ = 0.5 and the modal separation. As the synaptic perturbation becomes stronger, the height of the prior increases and the height of the mode corresponding to sensory evidence decreases (Fig. 9c, left panel). The perceptual estimate mean gradually shifts from the sensory evidence at **s**_1_ to the prior **s**_0_ (Fig. 9c, top right panel). The perceptual estimate variance also depends non-monotonically on the strength of synaptic perturbation (Fig. 9c, bottom right panel). This observation cannot be verified in the experimental data due to the limited data size and thus forms a testable prediction for future experiments.

## Discussion

In this study, we have presented a theory (i.e., FNS) of probabilistic neural computations through the illustration of its neural circuit implementation as well as the normative formulation of its computational properties. By extending existing models that are mainly based on temporally variable or fluctuating dynamics^13,14^, FNS exploits rich, complex fluctuations of neural population activity both in time and in space for efficiently performing sampling-based probabilistic computations; FNS thus offers an approach to addressing the long-standing challenge of sampling and representing multimodal distributions. Our probabilistic neural computation theory provides a unified account of a variety of findings on neural dynamics at the individual neuron and circuit levels as well as on perceptual phenomena such as perceptual switching and visual perception inference, thus establishing a framework for understanding neurophysiological and computational mechanisms of brain function.

Our FNS theory provides a new perspective on the role of complex spatiotemporal neural dynamics that exhibit large fluctuations across multiple scales, which are of a non-Gaussian (i.e., heavy-tailed) nature. Particularly, FNS-based probabilistic computations harness the power of the fractional Lévy motion of population activity patterns (i.e., neural ensembles); these patterns hover around one location for a while and then move or switch to another location in an intermittent manner, with their movement step sizes following heavy-tailed, power-law distributions. Such fractional motions in space give rise to irregular propagation trajectories and speeds with large variability. Propagating activity patterns have been widely observed at the circuit and the whole brain levels^22,23^; notably, localized gamma activity patterns with Lévy motion have been found in the MT area of marmoset monkeys^45^. We have repeated the analysis on the broadband LFP from ref. 45 (see Supplementary Information) and found that localized activity patterns of such broadband activity also show Lévy motion (Fig. S4). It has been shown that hippocampal sharp wave ripples exhibit movements with clusters of short step sizes that are intermittently interspersed by long jumps^46^. Nevertheless, to directly test our modeling prediction of propagating spiking patterns with Lévy motion, future studies need to focus on massive individual-neuron recordings and to analyze spiking patterns in the same way as done in our modeling study.

Another key property of sampling dynamics of FNS is that the autocorrelation function of the sample path exhibits an oscillatory component. Such an oscillatory component induces negative-going lobes in the autocorrelation; consequently, this would result in independent samples occurring in a much shorter time, thus playing an essential role in increasing the effective sampling speed as in the Hamiltonian Monte Carlo method^57,58^. In a previous modeling study^58^, such oscillatory activity arises from mapping Hamiltonian dynamics to the activity of excitatory and inhibitory neurons and its peak frequency is around 40 Hz (i.e., gamma oscillation). Importantly, it has been demonstrated that such gamma oscillations can naturally emerge in a circuit model trained for optimally performing sampling-based probabilistic inference^65^. In our model, as illustrated in the autocorrelation analysis of the sampling process, the temporal oscillation is in the theta range (3–8 Hz). We have found that the spike frequency adaptation is essential for the genesis of the theta oscillatory component, in accordance with other modeling studies^56^. However, different from oscillatory activity with clock-like periodicity in these models, neural oscillations in our model exhibit great variability and are accompanied by heavy-tailed, 1/f-like activity, as shown in the power spectrum of the firing rates of neural population activity. Notably, such temporal fluctuations with a 1/f component have been widely observed in neural population activity as recorded by LFP, EEG, and MEG during both spontaneous activity and task conditions^20^. Traditionally, such 1/f-like activity has been deemed unimportant and often removed from analyses to emphasize oscillatory components. In our theory, however, it is an integral part of our circuit implementation of FNS.

It has been found that theta oscillations are a hallmark feature of environmental sampling having now been linked to spatial attention sampling ^30,52^, eye movements in primates^66^, and whisking in rodents^49^. In some of these environmental sampling tasks, such as eye movement-based sampling of natural scenes, the existence of Lévy-like motion has been reported^67^. These observations lead us to predict that our FNS-based probabilistic computation might be the underlying computational mechanism of these cognitive functions; indeed, recently we have successfully applied FNS dynamics to explain key neural and behavioral effects of visual attention^51^. Furthermore, it is interesting to note that the phase of theta oscillations often modulates the amplitude of gamma fluctuations^68^. Such theta-gamma coupling suggests that it is important to explore whether and how each theta sampling cycle can be implemented through gamma activity, potentially unifying our FNS approach with the gamma dynamics-based sampling approaches^58,65^ and gaining a comprehensive understanding of probabilistic computations in neural circuits.

As formulated in the mathematical model with fractional order derivative and further illustrated in the spiking neural circuit, FNS exploiting the complex spatiotemporal dynamics mentioned above possesses some profound computational advantages. The sampling processes of FNS are quicker than those of the other methods such as the classical Langevin sampling method that has been implemented in stochastic recurrent neural networks. Importantly, FNS enables the efficient sampling of multimodal distributions even with far-away modes, due to the long jumps inherent to fractional movements of neural activity patterns. Sampling multimodal distributions is crucial for probabilistic computations, which, however, would not be possible in existing models, because they rely on Gaussian dynamics (i.e., Brownian motion) for implementing classical Langevin Monte Carlo sampling methods used in machine learning^69^. These methods are notorious for becoming trapped in a single mode, and lack the capacity to efficiently traverse through complex probability landscapes. Based on both the circuit and mathematical models, our theory of FNS makes a core prediction; that is, the mean exit time (i.e., the average time the sample path takes to leave one of the modes of a bimodal distribution) depends linearly on the modal separation. This prediction is falsifiable and hence represents a strong test of our FNS theory.

Our FNS can be implemented in biophysically realistic, two-dimensional spiking neural circuits incorporating two well-established properties of the cortex. One property is the dependence of synaptic connection probability on distance^36,37^ and another is the balance between excitatory and inhibitory inputs, with the former closely tracked by the latter^38^. Most previous models of sampling-based representations^14,15,58,59^, however, have focused on either normative probabilistic models or theories without specifying their underlying neural circuit mechanisms^14,15^, or on abstract neural circuits models ^58^, some of which have no separation of excitatory and inhibitory neurons thus violating Dale’s principle^59^. Networks with one-dimensional structure (i.e., a ring structure) have been trained to produce a fast sampling-based inference exploiting cortical-like temporal dynamics^65^. Since these networks do not intrinsically produce variable neural dynamics, an external source of fluctuations is required. In contrast, in our circuit model, spatiotemporal activity patterns with large fluctuations are intrinsically generated and the 2D spatial structure supports the emergence of such fluctuations. This scenario is similar to other complex physical systems where such a 2D spatial extension is important for the emergence of spatiotemporal patterns with complex dynamics^70^. In our model, the complex spatiotemporal patterns with fractional motions only emerge when the circuit works near the transition between different activity states; these findings are consistent with recent studies proposing that complex cortical dynamics could be better understood in the dynamical regime close to the transition between different cortical states (i.e., asynchronous and synchronous/coherent states)^71–73^, but go beyond them by revealing the fundamental functional role of complex spatiotemporal cortical dynamics in probabilistic neural computations.

The circuit mechanism of FNS also has important implications for constructing efficient artificial neural networks in machine learning. Large-scale artificial neural networks such as the Boltzmann machine^74^ and deep belief networks^75^ often perform complex computations through employing probabilistic sampling. Most of these artificial neural networks perform probabilistic sampling by using the classical MCMC driven by Gaussian noise, so they suffer from the similar problem as faced by the existing models of neural probabilistic sampling, in that these methods lack the ability to jump across low-probability regions and to traverse through the probability landscape (also known as the ‘mixing’ ability). The problem of mixing becomes even more pronounced when dealing with larger complex datasets. The common remedy for these methods relies on some variants of simulated tempering^76^, which change the temperature parameter in order to globally flatten the solution landscapes during the sampling process. These tempering methods, however, come with a cost of their own, because they require extra computations and parameter-tuning that assume knowledge about the global state of the artificial neural networks. However, our circuit-based FNS mechanism indicates that powerful mixing abilities for representing multimodal distributions can emerge from the circuits that are essentially locally coupled, and importantly this happens in a fundamentally autonomous manner without tuning any global parameters during the sampling process. Motivated by these properties of the circuit-implementation of FNS, we thus suggest that future large-scale, information processing neural network models may benefit from our circuit mechanism of FNS, if they are designed to exploit fractional Lévy-like diffusion and oscillations for sampling complex, high dimensional probability landscapes.

As we have demonstrated, the FNS-based probabilistic sampling can implement perceptual inference that explains both perceptual estimate statistics and the bimodality of perceptual estimate distributions as found in a recent study of motion direction estimation task^32^. Regarding probabilistic inference, our model makes two main predictions: first, the perceptual estimate variance is approximately a quadratic function of the modal separation between the sensory evidence and the prior; second, the perceptual estimate variance decreases with stimulus contrast but this trend is reversed when the contrast level is small. These model predictions are consistent with our reanalysis of the experimental data^32^, except for the cases when the stimulus contrast level is low; a conclusive test of these predictions will require more specific manipulation of stimuli to cover the low contrast range. Previously, a binary switching model with Gaussian noise was used to explain the bimodal property of perceptual estimates^32^. In our model, due to the non-equilibrium, fractional nature of Lévy motion, the activity patterns exhibit short-step clusters that are interspersed by long jumps, thus giving rise to clustering and switching-like behaviors and the resulting bimodality of the estimate probability distributions. Rather than being simplistic and binary, the switching-like behavior in our FNS model happens across multiple scales with large fluctuations, as evidenced by step sizes with heavy-tailed, power-law distributions. Switching-like dynamics with large fluctuations have indeed been found in the inferotemporal area of monkey during sensory processing^33^, in the orbitofrontal cortex^34^, and in the lateral intraparietal area^35^ during decision making. Our FNS mechanism of probability inference might therefore be generally applicable to understanding these key brain functions.

## Methods

### Spiking neural circuit implementation of FNS

The spatially extended spiking neural circuit model consists of *N*^E^ excitatory neurons and *N*^I^ inhibitory neurons embedded in a two-dimensional feature space with periodic boundary conditions^40^. Each neuron *i* is assigned to spatial coordinates **s**_*i*_ = (*x*_*i*_, *y*_*i*_) representing the preferred stimulus features (orientation and color) of that neuron, with both *x*_*i*_ and *y*_*i*_ ranging from −*π* to *π*. We consider *N*^E^ = 63 × 63 = 3969 excitatory neurons on a square grid and *N*^I^ = 1000 inhibitory neurons with uniformly random locations. The ratio between *N*^E^ and *N*^I^ is around 4. The Euclidean distance is calculated between each pair of neurons *i* from population *α* and *j* from population *β*, where *α* and *β* are either excitatory (E) or inhibitory (I). The connection probability between neurons is proportional to the distance-dependent factor ${{{\Omega }}}_{ij}^{\alpha \beta }={e}^{-{D}_{ij}^{\alpha \beta }/{\tau }_{D}^{\alpha \beta }}$. For the excitatory connections the spatial scales are ${\tau }_{D}^{{{{{{{{\rm{EE}}}}}}}}}=8$ and ${\tau }_{D}^{{{{{{{{\rm{IE}}}}}}}}}=10$ grid units, whereas for the inhibitory connections the spatial scales are ${\tau }_{D}^{{{{{{{{\rm{II}}}}}}}}}={\tau }_{D}^{{{{{{{{\rm{EI}}}}}}}}}=20$ grid units.

The subthreshold membrane potential ${V}_{i}^{\alpha }$ of neuron *i* in population *α* follows

5 $$C\frac{d{V}_{i}^{\alpha }(t)}{dt}=-{g}_{L}[{V}_{i}^{\alpha }(t)-{V}_{{{{{{{{\rm{L}}}}}}}}}]+{I}_{i,{{{{{{{\rm{K}}}}}}}}}^{\alpha }(t)+{I}_{i,{{{{{{{\rm{rec}}}}}}}}}^{\alpha }(t)+{I}_{i,{{{{{{{\rm{ext}}}}}}}}}^{\alpha }(t),$$

where the membrane capacitance *C* = 0.25 nF, the leak conductance *g*_*L*_ = 16.7 nS, and *V*_*L*_ = −70 mV is the reversal potential for the leak current. ${I}_{i,{{{{{{{\rm{K}}}}}}}}}^{\alpha }(t)$ is the potassium current, ${I}_{i,{{{{{{{\rm{rec}}}}}}}}}^{\alpha }(t)$ is the recurrent synaptic current received by the neuron and ${I}_{i,{{{{{{{\rm{ext}}}}}}}}}^{\alpha }(t)$ is the external current. When the membrane potential reaches the threshold *V*_th_ = − 50 mV, a spike is emitted and the membrane potential is reset to the potential *V*_rt_ = −60 mV for an absolute refractory period *τ*_f_ = 4 ms. The potassium current is given by ${I}_{i,{{{{{{{\rm{K}}}}}}}}}^{\alpha }(t)=-{g}_{i,{{{{{{{\rm{K}}}}}}}}}^{\alpha }(t)({V}_{i}^{\alpha }(t)-{V}_{{{{{{{{\rm{K}}}}}}}}})$, where ${g}_{i,{{{{{{{\rm{K}}}}}}}}}^{\alpha }(t)$ is the active potassium conductance and *V*_K_ = −85 mV. The dynamics of the potassium conductance are described by

6 $$\frac{d{g}_{i,{{{{{{{\rm{K}}}}}}}}}^{\alpha }(t)}{dt}=-\frac{{g}_{i,{{{{{{{\rm{K}}}}}}}}}^{\alpha }(t)}{{\tau }^{{{{{{{{\rm{K}}}}}}}}}}+{{\Delta }}{g}_{{{{{{{{\rm{K}}}}}}}}}\mathop{\sum}\limits_{k}\delta \big(t-{t}_{j,k}^{\alpha }\big),$$

where ${t}_{j,k}^{\alpha }$ is the time of the *k*th spike emitted by neuron *i* from population *α*, Δ*g*_K_ = 10 nS and *τ*^K^ = 80 ms. Because spike frequency adaptation has been primarily observed in cortical pyramidal neurons, we only include such adaptation for excitatory neurons in our model. The recurrent synaptic current ${I}_{i,{{{{{{{\rm{rec}}}}}}}}}^{\alpha }(t)$ in Eq. (5) is

7 $${I}_{i,{{{{{{{\rm{rec}}}}}}}}}^{\alpha }(t)=\mathop{\sum}\limits_{\beta }\Big[-{g}_{i}^{\alpha \beta }(t)\big({V}_{i}^{\alpha }-{V}_{{{{{{{{\rm{rev}}}}}}}}}^{\beta }\big)\Big],$$

where ${g}_{i}^{\alpha \beta }(t)$ is the conductance of the recurrent current from the presynaptic population *β*. The excitatory and inhibitory reversal potentials are ${V}_{{{{{{{{\rm{rev}}}}}}}}}^{{{{{{{{\rm{E}}}}}}}}}=0$ mV and ${V}_{{{{{{{{\rm{rev}}}}}}}}}^{{{{{{{{\rm{I}}}}}}}}}=-80$ mV, respectively. The conductance ${g}_{i}^{\alpha \beta }(t)$ is given by

8 $${g}_{i}^{\alpha \beta }(t)=\mathop{\sum }\limits_{j=1}^{{N}^{\beta }}{{a}}_{ij}^{\alpha \beta }{J}_{ij}^{\alpha \beta }{s}_{j}^{\beta }(t),$$

where ${a}_{ij}^{\alpha \beta }$ and ${J}_{ij}^{\alpha \beta }$ represent the coupling topology and the connection strength, respectively, as detailed in the original paper^40^. Specifically, the synaptic connection probability between neurons decreases in an exponential manner as distance between neurons increases, consistent with experimental findings^36,37^. The non-dimensional gating ${s}_{ij}^{\alpha \beta }(t)$ describes the synaptic dynamics

$$\frac{d{s}_{ij}^{\alpha \beta }(t)}{dt}=-\frac{{s}_{ij}^{\alpha \beta }(t)}{{\tau }_{{{{{{{{\rm{d}}}}}}}}}^{\alpha }}+\mathop{\sum}\limits_{k}{h}^{\beta }\left(t-{t}_{j,k}^{\beta }-{d}_{ij}^{\alpha \beta }\Big(1-{s}_{ij}^{\alpha \beta }(t)\Big)\right.,$$

9 $${h}^{\beta }(t)=\left\{\begin{array}{l}1/{\tau }_{{{{{{{{\rm{r}}}}}}}}}^{\beta },\,{{{{{{{\rm{if}}}}}}}} \; 0\le t\le {\tau }_{{{{{{{{\rm{r}}}}}}}}}^{\beta } \\ 0,\quad {{{{{{{\rm{otherwise}}}}}}}} \end{array}\right.,$$

where ${\tau }_{{{{{{{{\rm{d}}}}}}}}}^{\beta }$ and ${\tau }_{{{{{{{{\rm{r}}}}}}}}}^{\beta }$ are the decay and rise time constants, respectively, ${t}_{j,k}^{\beta }$ is the time point of the *k*th spike of neuron *j* from population *β*, and ${d}_{ij}^{\alpha \beta }$ is the conduction delay drawn from a uniform distribution between 0 and 4 ms.

In our model, we consider an essential neurophysiological feature of local cortical circuits, that is, the excitatory post-synaptic currents are proportional to the inhibitory ones, with a homogeneous ratio across all excitatory neurons, as found in mouse primary visual cortex layer 2/3^53^. To model this in our heterogeneous circuit, we consider the I–E ratio ${\xi }_{i}=\mathop{\sum }\nolimits_{k=1}^{{K}_{i,{{{{{{{\rm{in}}}}}}}}}^{{{{{{{{\rm{EI}}}}}}}}}}{J}_{jk}^{{{{{{{{\rm{EI}}}}}}}}}/{\sum }_{j}{J}_{ij}^{{{{{{{{\rm{EE}}}}}}}}}$, where ${K}_{i,{{{{{{{\rm{in}}}}}}}}}^{{{{{{{{\rm{EI}}}}}}}}}$ denotes the number of connections (in-degree) received by excitatory neuron *i* from the inhibitory population and the connection strength values ${J}_{ij}^{{{{{{{{\rm{EE}}}}}}}}}$ are determined by the reverse pooling method^40^. To equalize the I–E ratio *ξ*_*i*_ across the neurons to a desired network-wide ratio, that is, 〈*ξ*_*i*_〉 = *ξ*, the ${J}_{ij}^{{{{{{{{\rm{EI}}}}}}}}}$ for neuron *i* are sampled from a Gaussian distribution with a mean equal to $\xi {\sum }_{j}{J}_{ij}^{{{{{{{{\rm{EE}}}}}}}}}/{K}_{i,{{{{{{{\rm{in}}}}}}}}}^{{{{{{{{\rm{EI}}}}}}}}}$ and a standard deviation that is 25% of the mean. The I–E ratio *ξ* is varied as a system parameter for demonstrating the emergence of localized activity patterns with Lévy motion (Fig. 4) but is otherwise fixed at the value *ξ*_*c*_ = 3.4.

It has been shown previously that the circuit model exhibits a rich repertoire of dynamical activity states, ranging from asynchronous to localized and global propagating wave states^40^. Particularly, it has shown that around the phase transition between the asynchronous and localized propagating wave states, our circuit model exhibits nonlinear response properties different from the classical balanced state and can quantitatively reproduce a variety of major empirical findings regarding neural spatiotemporal dynamics. The phase transition was characterized by calculating the susceptibility and the branching parameter of spikes^40^. In the present study, we only change one parameter, the I–E ratio, to study the spatiotemporal properties (i.e., Lévy motion in space and theta oscillations in time) of the dynamical activity pattern emerging in this transition regime and their fundamental roles in probabilistic computations.

To study how the activity pattern samples non-trivial distributions, external stimuli are applied in the form of independent Poisson spike trains with specific firing rates. First, for sampling unimodal distributions, we apply an external feedforward input to each neuron *i* in the recurrent population as independent Poisson spikes whose firing rate is equal to

10 $${\lambda }_{i}={r}_{0}\left[1+c\exp \left(-\frac{\parallel {{{{{{{{\bf{s}}}}}}}}}_{i}-{{{{{{{{\bf{s}}}}}}}}}{*}{\parallel }^{2}}{2{\sigma }^{2}}\right)\right],$$

where *σ* = 0.6 is the width of the feedforward input, and *c* ≥ 0 is the contrast. When *c* = 0, the network receives the baseline background input with a uniform rate *r*_0_ = 0.85 kHz. The true value of the stimulus feature is fixed at s* = (0, 0) without loss of generality. Second, for sampling bimodal distribution, we apply an external feedforward input with firing rate equal to

11 $${\lambda }_{i}={r}_{0}\left[1+{c}_{1}\exp \left(-\frac{\parallel {{{{{{{{\bf{s}}}}}}}}}_{i}-{{{{{{{{\bf{s}}}}}}}}}_{1}{\parallel }^{2}}{2{\sigma }_{1}^{2}}\right)+{c}_{2}\exp \left(-\frac{\parallel {{{{{{{{\bf{s}}}}}}}}}_{i}-{{{{{{{{\bf{s}}}}}}}}}_{2}{\parallel }^{2}}{2{\sigma }_{2}^{2}}\right)\right].$$

The baseline firing rate is reduced to *r*_0_ = 0.5 kHz so that spontaneous activity (corresponding to when *c*_1_ = *c*_2_ = 0) is suppressed, ensuring a low-probability region between the modes of the sampled bimodal distribution. The true value of the stimulus feature s* is unknown by the observer and could be either of the alternative interpretations **s**_1_ or **s**_2_. We investigate the performance of our network in sampling bimodal distributions by varying the stimulus contrast levels *c*_1_ and *c*_2_ and the modal separation Δ**s** = ∥**s**_1_ − **s**_2_∥.

To embed prior in the recurrent synaptic connectivity, we apply a spatially heterogeneous perturbation to the excitatory-to-excitatory synaptic weight

12 $${{\Delta }}{J}_{ij}^{{{{{{{{\rm{EE}}}}}}}}}={c}_{0}\phi ({{{{{{{{\bf{s}}}}}}}}}_{i};{{{{{{{{\bf{s}}}}}}}}}_{0},d)\phi ({{{{{{{{\bf{s}}}}}}}}}_{j};{{{{{{{{\bf{s}}}}}}}}}_{0},d)$$

where **s**_*i*_ = (*x*_*i*_, *y*_*i*_) is the coordinates of neuron *i* in the feature space, *c*_0_ is the magnitude of the weight perturbation, and *ϕ* is a two-dimensional bell-shaped function

13 $$\phi ({{{{{{{\bf{s}}}}}}}};{{{{{{{{\bf{s}}}}}}}}}_{0},d)=\exp \Big[\frac{1}{{d}^{2}}(\cos (x-{x}_{0})+\cos (y-{y}_{0}))\Big],$$

centered at **s**_0_ = (*x*_0_, *y*_0_) with the width parameter *d*. In this study, we fix **s**_0_ = (0, 0) and *d* = 1.3.

### Calculating CoM, MSD, increment distributions, and neural correlations

Our spatially extended network exhibits localized spiking activity patterns with complex spatiotemporal dynamics. To characterize the dynamics of the pattern, we first track its center of mass (CoM), ${\hat{{{{{{{{\bf{s}}}}}}}}}}_{t}=({\hat{x}}_{t},{\hat{y}}_{t})$ based on the population vector of excitatory neurons^77^

14 $${\hat{x}}_{t}={{{{{{{\rm{Arg}}}}}}}}\mathop{\sum}\limits_{j\in E}{n}_{j}(t){e}^{i{x}_{j}},$$

where *x*_*j*_ ∈ [−*π*, *π*) is the *x*-coordinate of the *j*th excitatory neuron, *n*_*j*_ is the spike count over a small time window [*t* − *τ*, *t*) (here we set *τ* = 15 ms), *i* is the imaginary unit, and Arg is the principal complex argument in [−*π*, *π*). The *y*-coordinate of the CoM ${\hat{y}}_{t}$ is defined similarly. From a population coding point of view, Eq. (14) is also known as a complex estimator, which is equivalent to fitting a cosine function to the spike counts in the least-square sense^78^. In our study, the trajectory of the CoM of the activity pattern is the sample path of FNS.

Based on the trajectories of CoM, we next calculate the increments and the MSD. Specifically, the raw coordinates ${\hat{{{{{{{{\bf{s}}}}}}}}}}_{t}$ over the periodic space are first unwrapped to linear coordinates in order to accommodate distance calculations across the periodic boundary. The mean-squared displacement of the CoM is then ${{{{{{{\rm{MSD}}}}}}}}({{\Delta }}t)=\langle \parallel {\hat{{{{{{{{\bf{s}}}}}}}}}}_{t+{{\Delta }}t}-{\hat{{{{{{{{\bf{s}}}}}}}}}}_{t}{\parallel }^{2}\rangle$, where Δ*t* is the time lag. For fitting the increment distribution, we set Δ*T* = 15 ms to ensure no temporal correlation is artificially introduced due to overlaps in the spike count time window. The increments are then fitted to a symmetric Lévy stable distribution ${{{{{{{\mathcal{S}}}}}}}}\alpha {{{{{{{\mathcal{S}}}}}}}}(\alpha,\gamma )$ with the tail exponent *α* ∈ (0, 2] and the scale parameter *γ* > 0, as defined by Eq. (15). The parameters of the distribution are estimated with maximum likelihood. The MSD is averaged across 100 independent realizations of networks, each collecting 50 s of samples, whereas the increment histogram is produced with samples collected over 10 s, repeated across 5 random realizations of the network.

To characterize the state transition from the asynchronous to propagating wave state, we calculate local pairwise correlations of spike counts. We first divide the two-dimensional network into non-overlapping local patches of size 9 × 9 grid units and then calculate the pairwise spike count correlation within each patch. Finally, the mean local pairwise correlation is obtained by averaging the correlation coefficients of individual patches (49 patches in total). A weak local pairwise correlation indicates asynchronous activity without any structured patterns, whereas a strong local pairwise correlation indicates a spatially coherent wave pattern. The spike count time window is set to 100 ms with a total simulation time of 10 s repeated over 5 independent trials.

### Derivations of the mathematical model for FNS

To understand the algorithmic nature of FNS implemented by our spiking circuit model, we develop a mathematical model based on stochastic dynamics driven by Lévy motion, rather than Brownian motion as in the classical Langevin MCMC. We consider a class of Lévy motion ${L}_{t}^{\alpha }$ whose increments follow symmetric Lévy stable distributions, denoted as ${{{{{{{\mathcal{S}}}}}}}}\alpha {{{{{{{\mathcal{S}}}}}}}}(\alpha,\gamma )$, with a tail index *α* and a scale parameter *γ*. For clarity and analytical tractability, we restrict our discussion to the one-dimensional case. The probability density of ${{{{{{{\mathcal{S}}}}}}}}\alpha {{{{{{{\mathcal{S}}}}}}}}$ can be expressed as^28^

15 $$p(x)=\frac{1}{\pi }\int\nolimits_{0}^{\infty }\exp (-\gamma {\omega }^{\alpha })\cos (\omega x)d\omega .$$

The probability density exhibits a heavy tail, with power-law asymptote of the form^79^

16 $$p(x) \sim|x{|}^{-1-\alpha }\left[{\gamma }^{\alpha }\sin \Big(\frac{\pi \alpha }{2}\Big)\frac{{{\Gamma }}(\alpha+1)}{\pi }\right],$$

for all 0 < *α* < 2. Note that *α* = 1 corresponds to the special case of a Cauchy distribution and *α* = 2 corresponds to a Gaussian distribution. The increment ${{\Delta }}{L}_{t}^{\alpha }={L}_{t}^{\alpha }-{L}_{t^{\prime} }^{\alpha }$ with ${{\Delta }}t=t-t^{\prime} > \, 0$ follows ${{{{{{{\mathcal{S}}}}}}}}\alpha {{{{{{{\mathcal{S}}}}}}}}(\alpha,{{{\Delta }}t}^{\frac{1}{\alpha }})$.

To derive the mathematical model described by Eq. (2), we start with the general formula presented in ref. 80

17 $$d{{{{{{{{\bf{z}}}}}}}}}_{t}=({{{{{{{\bf{D}}}}}}}}+{{{{{{{\bf{Q}}}}}}}})b({{{{{{{\bf{z}}}}}}}})dt+{{{{{{{{\bf{D}}}}}}}}}^{1/\alpha }d{{{{{{{{\bf{L}}}}}}}}}_{t}^{\alpha },$$

where ${{{{{{{{\bf{z}}}}}}}}}_{{{{{{{{\bf{t}}}}}}}}}={({x}_{t},{v}_{t})}^{T}$, **D** is a positive semi-definite matrix describing the fractional diffusion, and **Q** is a skew-symmetric matrix describing the interplay between position *x*_*t*_ and momentum *v*_*t*_. The temporal evolution of the probability density *p*(**z**, *t*) is given by the fractional Fokker-Planck equation,

18 $${\partial }_{t}p({{{{{{{\bf{z}}}}}}}},t)=-{\partial }_{{{{{{{{\bf{z}}}}}}}}}({{{{{{{\bf{D}}}}}}}}+{{{{{{{\bf{Q}}}}}}}})b({{{{{{{\bf{z}}}}}}}})p({{{{{{{\bf{z}}}}}}}},t)-{{{{{{{\bf{D}}}}}}}}{{{{{{{{\mathcal{D}}}}}}}}}^{\alpha }p({{{{{{{\bf{z}}}}}}}},t),$$

where the fractional Riesz derivative ${{{{{{{{\mathcal{D}}}}}}}}}^{\alpha }$ is defined as

19 $${{{{{{{{\mathcal{D}}}}}}}}}^{\alpha }[f]={{{{{{{{\mathcal{F}}}}}}}}}^{-1}|{{{{{{{\bf{k}}}}}}}}{|}^{\alpha }{{{{{{{\mathcal{F}}}}}}}}[f],$$

through the Fourier transform ${{{{{{{\mathcal{F}}}}}}}}[f]=\int d{{{{{{{\bf{z}}}}}}}}{e}^{-i{{{{{{{\bf{k}}}}}}}}\cdot {{{{{{{\bf{z}}}}}}}}}f({{{{{{{\bf{z}}}}}}}})$. Fractional order derivatives generalize the notion of differentiation to fractional orders and are powerful mathematical tools for describing complex dynamics^81^; recently, fractional Fokker-Planck equations have been used to explain how non-Gaussian neural dynamics emerge from biologically realistic neural circuits^82^. It can be shown that the temporal evolution of *p*(**z**, *t*) converges to a stationary distribution $\pi ({{{{{{{\bf{z}}}}}}}})\propto \phi ({{{{{{{\bf{z}}}}}}}})=\exp (-H({{{{{{{\bf{z}}}}}}}}))=\exp (-U(x)-T(v))$, related to the drift term by

20 $$b({{{{{{{\bf{z}}}}}}}})=-\frac{{{{{{{{{\mathcal{D}}}}}}}}}^{\alpha -2}[\phi ({{{{{{{\bf{z}}}}}}}}){\partial }_{{{{{{{{\bf{z}}}}}}}}}H({{{{{{{\bf{z}}}}}}}})]}{\phi ({{{{{{{\bf{z}}}}}}}})}.$$

To retain both the efficient sampling provided by the Hamiltonian dynamics as well as the powerful mixing ability provided by the fractional dynamics, we set $${{{{{{{\bf{D}}}}}}}}=\big[\begin{array}{ll}\gamma &0\\ 0&0\end{array}\big]$$ and $${{{{{{{\bf{Q}}}}}}}}=\big[\begin{array}{ll}0&-\beta \\ \beta &0\end{array}\big]$$ and obtain

21 $$d{x}_{t} 	=-\gamma \frac{{{{{{{{{\mathcal{D}}}}}}}}}^{\alpha -2}[\phi ({{{{{{{\bf{z}}}}}}}}){\partial }_{x}U(x)]}{\phi ({{{{{{{\bf{z}}}}}}}})}dt+\beta \frac{{{{{{{{{\mathcal{D}}}}}}}}}^{\alpha -2}[\phi ({{{{{{{\bf{z}}}}}}}}){\partial }_{v}T(v)]}{\phi ({{{{{{{\bf{z}}}}}}}})}dt+{\gamma }^{1/\alpha }d{L}_{t}^{\alpha },\\ d{v}_{t} 	=-\beta \frac{{{{{{{{{\mathcal{D}}}}}}}}}^{\alpha -2}[\phi ({{{{{{{\bf{z}}}}}}}}){\partial }_{x}U(x)]}{\phi ({{{{{{{\bf{z}}}}}}}})}dt.$$

Although convergence to the stationary distribution is guaranteed mathematically, numerically simulating this set of equations is challenging since there are no straightforward ways for evaluating the Riesz fractional derivative with two dimensions. To overcome this numerical challenge, we exploit the fact that we are only interested in sampling the target distribution of *π*(*x*) instead of the joint stationary distribution of *π*(*x*, *v*) and make two simplifications as follows. First, we approximate the Riesz fractional derivative ${{{{{{{{\mathcal{D}}}}}}}}}^{\alpha }$ with its partial versions ${{{{{{{{\mathcal{D}}}}}}}}}_{x}^{\alpha }$ and ${{{{{{{{\mathcal{D}}}}}}}}}_{v}^{\alpha }$, such that

22 $$d{x}_{t} 	=-\gamma \frac{{{{{{{{{\mathcal{D}}}}}}}}}_{x}^{\alpha -2}[\phi (x){\partial }_{x}U(x)]}{\phi (x)}dt+\beta \frac{{{{{{{{{\mathcal{D}}}}}}}}}_{v}^{\alpha -2}[\psi (v){\partial }_{v}T(v)]}{\psi (v)}dt+{\gamma }^{1/\alpha }d{L}_{t}^{\alpha },\\ d{v}_{t} 	=-\beta \frac{{{{{{{{{\mathcal{D}}}}}}}}}_{x}^{\alpha -2}[\phi (x){\partial }_{x}U(x)]}{\phi (x)}dt.$$

The partial Riesz fractional derivative can then be effectively evaluated using a fractional centered difference scheme^83,84^. We find that this approximation in practice provides the correct sample distribution through numerical simulation. Second, by setting $\psi (v)=\exp (-T(v))$ to be a symmetric *α*-stable distribution *SαS*(1/*α*), it holds that

23 $$\frac{{{{{{{{{\mathcal{D}}}}}}}}}_{v}^{\alpha -2}[\psi (v){\partial }_{v}T(v)]}{\psi (v)}=v.$$

By applying these two simplifications, we obtain the fractional Hamiltonian dynamics for modeling fractional neural sampling (Eq. (2)). As we show numerically in Results, the distribution of the samples generated by Eq. (2) converges to the target distribution *π*(*x*). For all simulations we use the Euler-Maruyama scheme with a step size of Δ*t* = 0.001 with *γ* = 1, and apply a clipping $\tilde{b}(x)\approx \min (b(x),{b}_{\max })$ with ${b}_{\max }=500$ to avoid numeric overflow of the Riesz derivative near low-probability regions.

A number of properties about the Riesz derivative are worth noting. First, when *α* = 2, the Riesz fractional derivative coincides with the ordinary second-order derivative with a change of sign, that is, ${{{{{{{{\mathcal{D}}}}}}}}}^{2}=-{\partial }_{xx}-{\partial }_{vv}$. Second, unlike integer-order derivatives, the Riesz derivative cannot be broken down into the sum of the partial derivatives in each of its dimension, that is, ${{{{{{{{\mathcal{D}}}}}}}}}^{\alpha }\, \ne \, {{{{{{{{\mathcal{D}}}}}}}}}_{x}^{\alpha }+{{{{{{{{\mathcal{D}}}}}}}}}_{v}^{\alpha }$, as the right-hand side of this equation cannot preserve isotropy in the space spanned by (*x*, *v*).

### Exit time calculation for sampling processes of FNS

To characterize the ability of FNS for sampling bimodal distributions, we calculate how the mean exit time, which measures the average duration the sampler spends near one of the modes, changes as a function of modal separation. Suppose that the two modes of a bimodal distribution are centered at **s**_1_ and **s**_2_, then the exit time *τ*_exit_ from the first mode to the second mode can be defined as the duration the sample trajectory remains closer to **s**_1_ than to **s**_2_

24 $${\tau }_{{{{{{{{\rm{exit}}}}}}}}}=\inf \{t\ge 0:\parallel {\hat{{{{{{{{\bf{s}}}}}}}}}}_{t}-{{{{{{{{\bf{s}}}}}}}}}_{1}\parallel > \parallel {\hat{{{{{{{{\bf{s}}}}}}}}}}_{t}-{{{{{{{{\bf{s}}}}}}}}}_{2}\parallel \},$$

where the ${\hat{{{{{{{{\bf{s}}}}}}}}}}_{0}$ is any point satisfying $\parallel {\hat{{{{{{{{\bf{s}}}}}}}}}}_{{0}_{+}}-{{{{{{{{\bf{s}}}}}}}}}_{1}\parallel \le \parallel {\hat{{{{{{{{\bf{s}}}}}}}}}}_{{0}_{-}}-{{{{{{{{\bf{s}}}}}}}}}_{2}\parallel$ with ${\hat{{{{{{{{\bf{s}}}}}}}}}}_{{0}_{\pm }}$ denoting the one-sided limits $\mathop{\lim }\nolimits_{t\to \pm 0}{\hat{{{{{{{{\bf{s}}}}}}}}}}_{t}$. The mean exit time is then calculated as the average of all exit times in a sample trajectory. This definition applies to both the 1D mathematical model and the 2D neural circuit implementation. For the mathematical model, we use an open boundary condition rather than a periodic boundary condition in order to highlight the impact of modal separation on sampling. For the case of periodic boundary condition, the exit time increases similarly for small modal separations but saturates for large modal separations.

In the context of perceptual bistability, the exit time can be interpreted as perceptual dominance duration which is known to follow a right-skewed distribution. We fit the exit time calculated from the neural circuit model to two candidate distributions using the method of maximum likelihood. The first one is the Burr distribution with probability density function

25 $$p(x;c,\, k,\, \lambda )=\frac{ck}{\lambda }{\left(\frac{x}{\lambda }\right)}^{c-1}{\left[1+{\left(\frac{x}{\lambda }\right)}^{c}\right]}^{-k-1},$$

with parameters found to be *λ* = 65.0 ms (95% CI 62.7–65.3), *c* = 8.05 (95% CI 7.58–8.55), and *k* = 0.528 (95% CI 0.476–0.586). The second one is the gamma distribution with probability density

26 $$p(x;k,\, \theta )=\frac{1}{{{\Gamma }}(k){\theta }^{k}}{x}^{k-1}{e}^{-\frac{x}{\theta }},$$

with parameters found to be *k* = 10.4 (95% CI 9.9–10.9) and *θ* = 7.51 ms (95% CI 7.14–7.88). Note that the former features a power-law tail (with an exponent equal to −1 − *ck*) whereas the latter features a faster decaying exponential tail. Outliers (dominance duration <29 ms) are occasionally produced during the asynchronous phase of the spiking pattern and thus are omitted for this analysis.

### Analysis of a neural field model

To gain theoretical insights into how the drift term and sampled distribution are related to sensory input and synaptic weights, we consider a continuous neural field model^85^, which captures some features of the spiking circuit model, including the distance-dependent synaptic coupling and a localized activity pattern (bump activity) that moves randomly in the presence of noise. The continuous neural field model with one spatial dimension is described as

27 $$\tau \frac{\partial u}{\partial t}=-u+\int J(x,\, x^{\prime} )F[u](x^{\prime} \!,\, t)\rho dx^{\prime}+I(x),$$

where *u*(*x*, *t*) represents the synaptic current, $J(x \!,\, x^{\prime} )$ is the synaptic coupling function, *ρ* is the neural density, *I*(*x*) is the feedforward input current, and *F* is a quadratic neural activation with divisive normalization. We consider the following form of synaptic coupling $J(x \!,\, x^{\prime} )=\bar{J}(x-x^{\prime} )+{c}_{0}\tilde{J}(x \!,\, x^{\prime} ;{s}_{0})$, where $\bar{J}$ is a translation-invariant synaptic coupling function and $\tilde{J}$ is a heterogeneous synaptic perturbation centered around *s*_0_, and *c*_0_ is the strength of the perturbation. Similarly, the feedforward input is $I(x)=\bar{I}+{c}_{1}\tilde{I}(x;{s}_{1})+\xi (x),$ where $\bar{I}$ is a constant background input, $\tilde{I}(x;{s}_{1})$ is a stimulus-dependent feedforward input centered at *s*_1_, *c*_1_ represents stimulus contrast, and *ξ*(*x*) is an additive noise; see Supplementary Information for the detailed definitions of these terms. By applying a projection method, we obtain an explicit analytical expression for the drift term

28 $$\gamma b(x)=-{c}_{1}\frac{2\sqrt{2}{a}^{2}{d}_{1}}{\tau {({a}^{2}+{d}_{1}^{2})}^{3/2}}(x-{s}_{1})\exp \left[-\frac{{(x-{s}_{1})}^{2}}{4({a}^{2}+{d}_{1}^{2})}\right] \\ -{c}_{0}{r}_{0}\frac{4\sqrt{\pi }{a}^{3}{d}_{0}^{2}}{\tau {({a}^{2}+{d}_{0}^{2})}^{2}}(x-{s}_{0})\exp \left[-\frac{{(x-{s}_{0})}^{2}}{\frac{4}{3}({a}^{2}+{d}_{0}^{2})}\right],$$

where *a* is the width of the homogeneous synaptic coupling, *d*_1_ is the width of the feedforward input, *d*_0_ is the width of the synaptic perturbation, and *r*_0_ is the height of the localized activity. The corresponding sampled distribution is bimodal when *s*_1_ and *s*_0_ are far apart, in which case we can apply Laplace approximation to cast it into the form of a Gaussian mixture

29 $$p(x)\approx \frac{{w}_{1}}{{w}_{1}+{w}_{0}}g(x;{s}_{1} \!,\, {\kappa }_{1}/{c}_{1})+\frac{{w}_{0}}{{w}_{1}+{w}_{0}}g(x;{s}_{0},\, {\kappa }_{0}/{c}_{0}),$$

where *g*(*x*; *μ*, *σ*^2^) denotes a Gaussian distribution with mean *μ* and variance *σ*^2^. The variance of each of the modes is inversely proportional to the strength of perturbation *c*_1_ or *c*_0_, with the constant of proportionality equal to ${\kappa }_{1}=\frac{\gamma {c}_{\alpha }\tau {({a}^{2}+{d}_{1}^{2})}^{3/2}}{2\sqrt{2}{a}^{2}{d}_{1}}$, or ${\kappa }_{0}=\frac{\gamma {c}_{\alpha }\tau {({a}^{2}+{d}_{0}^{2})}^{2}}{4\sqrt{\pi }{a}^{3}{d}_{0}^{2}{r}_{0}}$, respectively. The mixture proportion is determined by ${w}_{1}=\sqrt{2\pi {\kappa }_{1}/{c}_{1}}\exp \Big(\frac{{c}_{1}}{\gamma {c}_{\alpha }\tau }\frac{4\sqrt{2}{a}^{2}{d}_{1}}{{({a}^{2}+{d}_{1}^{2})}^{1/2}}\Big)$ and ${w}_{0}=\sqrt{2\pi {\kappa }_{0}/{c}_{0}}\exp \big(\frac{{c}_{0}}{\gamma {c}_{\alpha }\tau }\frac{8\sqrt{\pi }{a}^{3}{d}_{0}^{2}{r}_{0}}{3({a}^{2}+{d}_{0}^{2})}\big)$. See Supplementary Information for the details of the mathematical derivation of Eq. (28) and Eq. (29).

### Reporting summary

Further information on research design is available in the Nature Research Reporting Summary linked to this article.

## Supplementary information

Supplementary Information

Reporting Summary

## Data Availability

The local field potential data used in this study are available on Zenodo^86^ (10.5281/zenodo.6806648). Source data are provided with this paper.

## Source data

Source Data

## Code availability

The code for simulations and analyses of the spiking neural circuit model^87^ (10.5281/zenodo.6806437) and the code for fractional Hamiltonian Monte Carlo sampling^88^ (10.5281/zenodo.6799461) are available without restrictions on Github.
