# Supplementary material for: Fractional neural sampling as a theory of spatiotemporal probabilistic computations in neural circuits
Source: Nat Commun. 2022 Aug 5;13:4572. doi: 10.1038/s41467-022-32279-z (PMC9356069; doi:10.1038/s41467-022-32279-z)
Supplement: Supplementary file 1 — Supplementary Information [file 41467_2022_32279_MOESM1_ESM.pdf]

Supplementary Information

Fractional neural sampling as a theory of spatiotemporal probabilistic computations in neural circuits

Yang Qi[1,2,3,4], Pulin Gong[1,*]

1 School of Physics, University of Sydney, NSW 2006, Australia
2 Institute of Science and Technology for Brain-Inspired Intelligence, Fudan University, Shanghai 200433, China
3 Key Laboratory of Computational Neuroscience and Brain-Inspired Intelligence (Fudan University), Ministry of Education, China
4 MOE Frontiers Center for Brain Science, Fudan University, Shanghai 200433, China

* *pulin.gong@sydney.edu.au*

1 Analysis of a neural field model

The neural field model with one spatial dimension is described as

$$\tau\frac{\partial u}{\partial t} = -u + \int J(x,x')F[u](x',t)\rho dx' + I(x), \tag{S1}$$

where $u(x,t)$ represents the synaptic current, F is the neural activation function, and $I(x)$ is the feedforward input. The neural activation function takes the form

$$F[u](x,t) = \frac{u^2(x,t)}{1+k\rho\int u(x',t)^2dx'}, \tag{S2}$$

where ρ the density of neurons and k represents the strength of global inhibition. When the synaptic weight $J(x,x')$ is translation-invariant and $I(x)$ is spatially uniform, there exists a stationary bump solution $u(x,t) = \bar{u}(x-\hat{x}_t)$ which is symmetric around its center of mass (CoM) $\hat{x}_t$. The stationary solution thus forms a continuous manifold parameterized by $\hat{x}$. It has been shown how feedforward input can modulate the dynamics of $\hat{x}_t$ through a projection method[85]. Here we extend this method to analyze the effect of spatially heterogeneous perturbation of synaptic weights. To implement prior into the recurrent synaptic connections, we impose a perturbation to the synaptic weight

$$J(x,x') = \bar{J}(x-x') + c_0\tilde{J}(x,x'), \tag{S3}$$

where $\bar{J}(x) = \frac{A}{\sqrt{2\pi}a}\exp(-\frac{x^2}{2a^2})$ and $\tilde{J}$ is a spatially inhomogeneous weight perturbation. Similarly, sensory evidence is modeled as a perturbation to the feedforward input as

$$I(x) = \bar{I} + c_1\tilde{I}(x) + \xi, \tag{S4}$$

where c_1 is the input contrast and $\bar{I}=0$. The input current and synaptic weight perturbation take the form

$$\tilde{I}(x;s_1) = u_0\exp\left[-\frac{(x-s_1)^2}{4d_1^2}\right], \tag{S5}$$

and

$$\tilde{J}(x,x';s_0) = u_0 \exp\left[-\frac{(x-s_0)^2}{4d_0^2}\right]\exp\left[-\frac{(x'-s_0)^2}{2d_0^2}\right], \tag{S6}$$

respectively. The corresponding stationary bump solution when $c_0 = c_1 = 0$ is

$$\bar{u}(x-\hat{x}_t) = u_0 \exp\left[-\frac{(x-\hat{x}_t)^2}{4a^2}\right], \tag{S7}$$

$$F[\bar{u}](x-\hat{x}_t) = r_0 \exp\left[-\frac{(x-\hat{x}_t)^2}{2a^2}\right], \tag{S8}$$

with $u_0 = [1+\sqrt{1-k/k_c}]A/(4\sqrt{\pi}ak)$ and $r_0 = [1+\sqrt{1-k/k_c}]/(2\sqrt{2\pi}ak\rho)$. To facilitate further analysis, we assume that the amplitude c_0 of the weight perturbation as well as the input contrast c_1 are small such that changes in $\bar{u}$ are negligible, leading to the approximation

$$\tau\frac{\partial\bar{u}}{\partial t} = -\bar{u} + \int \bar{J}(x-x')F[\bar{u}](x')dx' + \bar{I} + c_0\int \tilde{J}(x,x')F(\bar{u})dx' + c_1\tilde{I}(x) + \xi. \tag{S9}$$

To obtain an equation governing the dynamics of the CoM $\hat{x}_t$ of the bump activity, define the following projection operator

$$\langle v, f\rangle = \int v(x|\hat{x})f(x|\hat{x})dx, \tag{S10}$$

where v is the neutrally stable left eigenmode of the system computed at the stationary bump solution centered at $\hat{x}$

$$v(x-\hat{x}) = \frac{2a}{u_0\sqrt{(2\pi)^{1/2}a}}\frac{\partial\tilde{u}(x-\hat{x})}{\partial\hat{x}} = \frac{1}{\sqrt{(2\pi)^{1/2}a}}\frac{x-\hat{x}}{a}\exp\left[-\frac{(x-\hat{x})^2}{4a^2}\right]. \tag{S11}$$

Note the projection satisfies the following orthonormality properties $\langle v, v\rangle = 1$ and $\langle v, \bar{u}\rangle = 0$. Apply the projection operator to both sides of Eq. S9. For the left-hand side, we have

$$\langle v, \tau\partial_t\bar{u}\rangle = -\tau\langle v, \bar{u}'\rangle\frac{d\hat{x}_t}{dt} = \frac{\tau u_0\sqrt{(2\pi)^{1/2}a}}{2a}\langle v, v\rangle\frac{d\hat{x}_t}{dt} = \frac{\tau u_0\sqrt{(2\pi)^{1/2}a}}{2a}\frac{d\hat{x}_t}{dt}. \tag{S12}$$

For the right-hand side, the first three terms corresponding to the unperturbed system are canceled out. The projection of the term involving the recurrent weight perturbation is

$$\begin{aligned}\langle v, \textstyle\int \tilde{J}(x,x')F[\bar{u}](x')dx'\rangle &= -u_0\sqrt{(2\pi)^{1/2}a}\tfrac{\sqrt{2}ad_0}{(a^2+d_0^2)^{3/2}}(\hat{x}-s_0)\exp\left[-\frac{(\hat{x}-s_0)^2}{4(a^2+d_0^2)}\right]\\ &\quad\times r_0\sqrt{2\pi}\tfrac{ad_0}{\sqrt{a^2+d_0^2}}\exp\left[-\frac{(\hat{x}-s_0)^2}{2(a^2+d_0^2)}\right]\\ &= -u_0r_0\sqrt{(2\pi)^{1/2}a}\tfrac{2\sqrt{\pi}a^2d_0^2}{(a^2+d_0^2)^2}(\hat{x}-s_0)\exp\left[-\frac{(\hat{x}-s_0)^2}{\frac{4}{3}(a^2+d_0^2)}\right],\end{aligned} \tag{S13}$$

whereas that for the heterogeneous feedforward input is

$$\langle v, \tilde{I}\rangle = -u_0\sqrt{(2\pi)^{1/2}a}\tfrac{\sqrt{2}ad_1}{(a^2+d_1^2)^{3/2}}(\hat{x}-s_1)\exp\left[-\frac{(\hat{x}-s_1)^2}{4(a^2+d_1^2)}\right]. \tag{S14}$$

Since the focus here is to obtain the drift term, we do not explicitly specify the form of the noise ξ but simply assume that the projection of ξ satisfies

$$\langle v, \xi dt^{1/\alpha}\rangle = (\tfrac{\tau u_0\sqrt{(2\pi)^{1/2}a}}{2a}\gamma)^{1/\alpha}dL^\alpha. \tag{S15}$$

Finally, combining these results we arrive at the equation governing the dynamics of the CoM $\hat{x}_t$ of the bump activity,

$$d\hat{x}_t = \gamma b(\hat{x}_t)dt + \gamma^{1/\alpha}dL^\alpha, \tag{S16}$$

with

$$\gamma b(\hat{x}) = -c_1 \tfrac{2\sqrt{2}a^2 d_1}{\tau(a^2+d_1^2)^{3/2}}(\hat{x}-s_1)\exp\left[-\frac{(\hat{x}-s_1)^2}{4(a^2+d_1^2)}\right] - c_0 r_0 \tfrac{4\sqrt{\pi}a^3 d_0^2}{\tau(a^2+d_0^2)^2}(\hat{x}-s_0)\exp\left[-\frac{(\hat{x}-s_0)^2}{\frac{4}{3}(a^2+d_0^2)}\right]. \tag{S17}$$

Finally, we wish to obtain an explicit expression of the sampled distribution. To achieve this, we use the following approximation for the Riesz derivative $\mathcal{D}^{\alpha-2}f(x) \approx c_\alpha f(x)$ with $c_\alpha = \Gamma(\alpha-1)/\Gamma(\alpha/2)$[83] to obtained a simplified relationship

$$b(\hat{x}) \approx c_\alpha \partial_{\hat{x}} \log \phi(\hat{x}), \tag{S18}$$

between the drift $b(\hat{x})$ and the unnormalized sample distribution $\phi(\hat{x})$. This leads to

$$\begin{aligned}\phi(\hat{x}) &\approx \exp[\tfrac{1}{c_\alpha}\textstyle\int_{-\infty}^{\hat{x}} b(x')dx'] \\ &= \exp\left[\tfrac{c_1}{\gamma c_\alpha \tau}\tfrac{4\sqrt{2}a^2 d_1}{(a^2+d_1^2)^{1/2}}\exp[-\tfrac{(\hat{x}-s_1)^2}{4(a^2+d_1^2)}] + \tfrac{c_0}{\gamma c_\alpha \tau}\tfrac{8\sqrt{\pi}a^3 d_0^2 r_0}{3(a^2+d_0^2)}\exp[-\tfrac{(\hat{x}-s_0)^2}{\frac{4}{3}(a^2+d_0^2)}]\right].\end{aligned} \tag{S19}$$

This is a bimodal distribution when s_0 and s_1 are far apart, in which case we can apply Laplace approximation near each mode to further simplify the expression. When $\hat{x} \to s_1$, we have

$$\phi(\hat{x}) \approx \tfrac{w_1}{\sqrt{2\pi\kappa_1/c_1}}\exp\left[-\tfrac{(\hat{x}-s_1)^2}{2\kappa_1/c_1}\right], \tag{S20}$$

where $w_1 = \sqrt{2\pi\kappa_1/c_1}\exp\left(\tfrac{c_1}{\gamma c_\alpha \tau}\tfrac{4\sqrt{2}a^2 d_1}{(a^2+d_1^2)^{1/2}}\right)$ and $\kappa_1 = \tfrac{\gamma c_\alpha \tau(a^2+d_1^2)^{3/2}}{2\sqrt{2}a^2 d_1}$. This result shows that for sufficiently large contrast c_1, the variance of this mode is inversely proportional to c_1. Additionally, as the input width d_1 increases, the coefficient of proportionality κ_1 first decreases and then increases, with a minimum at $d_0 = a/\sqrt{2}$. Similarly, when $\hat{x} \to s_0$, we have

$$\phi(\hat{x}) \approx \tfrac{w_2}{\sqrt{2\pi\kappa_0/c_0}}\exp\left[-\tfrac{(\hat{x}-s_0)^2}{2\kappa_0/c_0}\right], \tag{S21}$$

where $w_0 = \sqrt{2\pi\kappa_0/c_0}\exp\left(\tfrac{c_0}{\gamma c_\alpha \tau}\tfrac{8\sqrt{\pi}a^3 d_0^2 r_0}{3(a^2+d_0^2)}\right)$ and $\kappa_0 = \tfrac{\gamma c_\alpha \tau(a^2+d_0^2)^2}{4\sqrt{\pi}a^3 d_0^2 r_0}$. Similar to the previous case, the variance of this mode is inversely proportional to the strength of synaptic perturbation c_0, and the coefficient of proportionality κ_0 first decreases and then increases with d_0, with a minimum at $d_0 = a$. Combine these two terms we obtain the normalized, Laplace-approximated sample distribution in the form of a Gaussian mixture

$$p(\hat{x}) \approx \tfrac{w_0}{w_0+w_1}g(\hat{x}; s_0, \kappa_0/c_0) + \tfrac{w_1}{w_0+w_1}g(\hat{x}; s_1, \kappa_1/c_1), \tag{S22}$$

where $g(\hat{x}; \mu, \sigma^2)$ denotes a Gaussian distribution with mean μ and variance σ^2. This approximation allows us to obtain simple expressions for the overall mean and variance of the bimodal distribution

$$\mathbb{E}[\hat{x}] = \tfrac{w_1}{w_1+w_0}s_1 + \tfrac{w_0}{w_1+w_0}s_0, \tag{S23}$$

and

$$\mathrm{Var}[\hat{x}] = \tfrac{w_1 w_0}{w_1+w_0}(s_1-s_0)^2 + \tfrac{w_1}{w_1+w_0}\tfrac{\kappa_1}{c_1} + \tfrac{w_0}{w_1+w_0}\tfrac{\kappa_0}{c_0}, \tag{S24}$$

which explain the linear and quadratic dependence of perceptual estimate mean and variance on modal separation, respectively, as observed in the spiking neural circuit model. The following parameter values are used in this study: $A = 0.05$, $a = 0.3$, $\rho = 10$, $k/k_c = 0.5$, $d_0 = \sqrt{5}a$, $d_1 = a$, $\tau = 1$, $\gamma = 0.1$, $\alpha = 1.5$.

2 Lévy motion of localized patterns found in broadband local field potential

Localized activity patterns following Lévy motion have been found in the gamma-band local field potential (LFP) recorded using multi-electrode arrays (10×10 electrodes) in area MT of marmoset monkeys[45]. Here, we repeat this analysis with the broadband LFP (0.1-500 Hz). We first standardize the LFP of each time frame by subtracting the mean and dividing by the standard deviation; as shown in Fig. S4a, the broadband activities are organized as spatially localized patterns. To detect such localized patterns, we apply a threshold of one standard deviation. We then trace the motion of the largest localized pattern of each time frame over time based on its spatiotemporal continuity (see ref.[45] for details of the algorithm). Only patterns with a minimum area of 4 recording sites and a duration greater than 0.5 s are considered; choices near these values yield similar results. We detect a total number of 204 localized patterns from the LFP recording over a time period of 300 s, with each pattern persisting for up to 5.3 s. A snapshot of the broadband LFP exhibiting localized activity pattern is shown in Fig. S4a, overlaid with its center of mass (CoM; circle) and trajectory (solid line) over the previous 100 ms. As in our modeling study, we calculate the increments of the CoM trajectory over 1 ms time intervals, and find that the resulting distribution can be fitted to a Lévy stable distribution with a tail index $\alpha = 1.38$ (Fig. S4b), indicating that localized pattern in the broadband LFP exhibits Lévy motion.

3 Supplementary figures

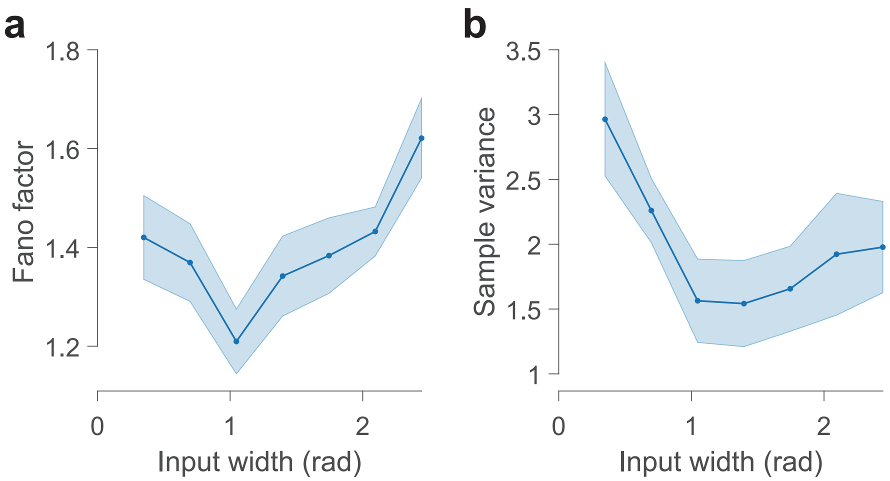

Figure S1. Neural response uncertainty and sample variance depend on input width. a Fano factor of spike counts for varying widths of the feedforward input, averaged over 20 trials of independently generated circuits. Shades indicates the standard error of the mean. **b** Sample variance for varying contrast levels of the feedforward input; shades indicate the standard deviation of the sample variance across 20 trials.

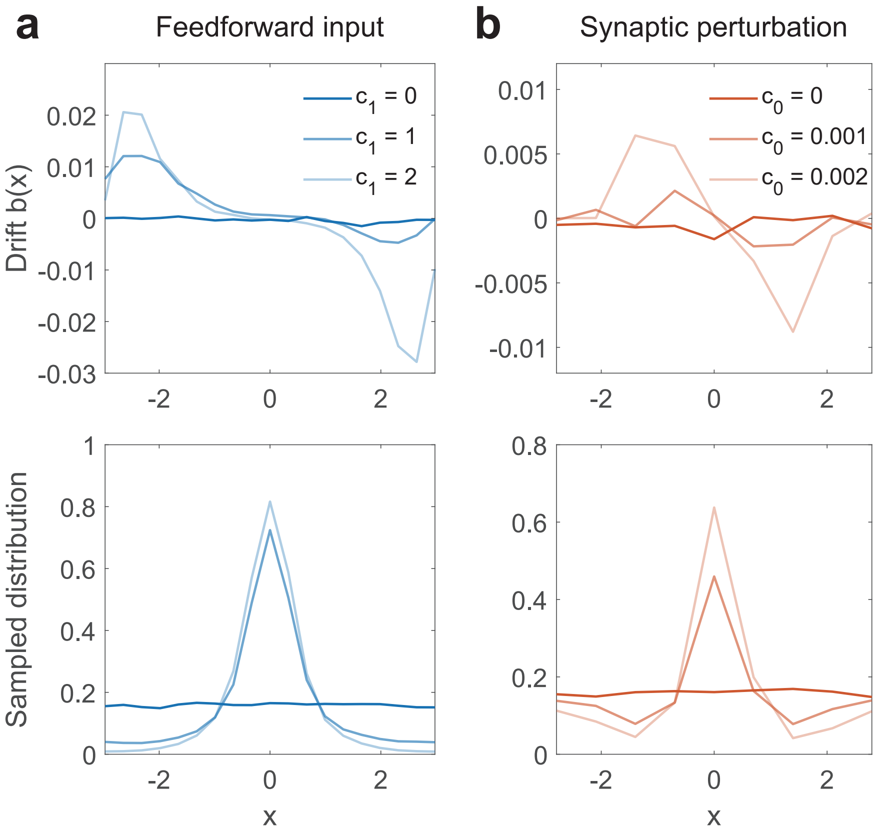

Figure S2. Empirical drift computed from the spiking neural circuit model. a Drift term (upper panel) and sampled distribution (lower panel) when the neural circuit receives a feedforward input with varying contrast levels c_1. **b** Drift term (upper panel) and sampled distribution (lower panel) when the neural circuit receives synaptic weight perturbation with varying strengths c_0. In each case, the localized activity pattern experiences a drift towards the center of the feedforward input or that of the synaptic perturbation, resulting in a unimodal sampled distribution.

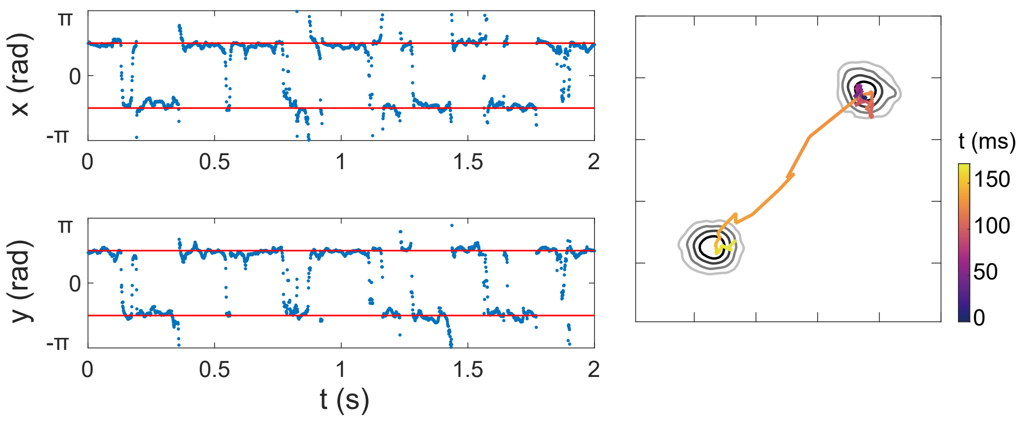

Figure S3. Localized activity pattern sampling a bimodal distribution in the spiking neural circuit without adaptation. Left panel: center-of-mass (CoM) trajectory (dots) of the localized pattern switches rapidly between two modes (red lines). Right panel: contour plot of the log sample probability density with a segment of the sample path overlaid on top.

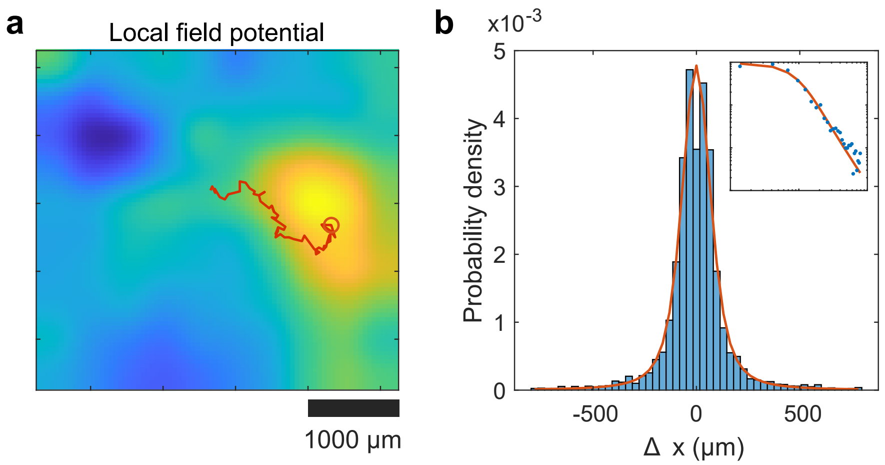

Figure S4. Localized activity pattern in broadband local field potentials exhibiting Lévy motion. a Broadband local field potentials recorded using a 10 x 10 electrode array exhibits a localized activity pattern. Circle: center of mass (CoM); solid line: trajectory of the localized pattern. **b** Histogram of the increment of the CoM is fitted to a Lévy stable distribution (solid line) with a tail index $\alpha = 1.38$. Inset: log-log plot of the tail of the increment distribution.
